# Two Novel Amyloid Proteins, RopA and RopB, from the Root Nodule Bacterium *Rhizobium leguminosarum*

**DOI:** 10.3390/biom9110694

**Published:** 2019-11-04

**Authors:** Anastasiia O. Kosolapova, Mikhail V. Belousov, Anna I. Sulatskaya, Maria E. Belousova, Maksim I. Sulatsky, Kirill S. Antonets, Kirill V. Volkov, Anna N. Lykholay, Oksana Y. Shtark, Ekaterina N. Vasileva, Vladimir A. Zhukov, Alexandra N. Ivanova, Pavel A. Zykin, Irina M. Kuznetsova, Konstantin K. Turoverov, Igor A. Tikhonovich, Anton A. Nizhnikov

**Affiliations:** 1 Laboratory for Proteomics of Supra-Organismal Systems, All-Russia Research Institute for Agricultural Microbiology (ARRIAM), 196608 St. Petersburg, Russia; kosolapova97@mail.ru (A.O.K.); belousovmix@gmail.com (M.V.B.); coryphella@mail.ru (M.E.B.); k.antonets@arriam.ru (K.S.A.); 2 Faculty of Biology, St. Petersburg State University (SPbSU), 199034 St. Petersburg, Russia; evasilieva@arriam.ru (E.N.V.); pavel.zykin@gmail.com (P.A.Z.); i.tikhonovich@spbu.ru (I.A.T.); 3 Laboratory of Structural Dynamics, Stability and Folding of Proteins, Institute of Cytology of the Russian Academy of Sciences, 194064 St. Petersburg, Russia; ansul@mail.ru (A.I.S.); imk@incras.ru (I.M.K.); kkt@incras.ru (K.K.T.); 4 Laboratory of Cell Morphology, Institute of Cytology of the Russian Academy of Sciences, 194064 St. Petersburg, Russia; m_sulatsky@mail.ru; 5 Research Resource Center “Molecular and Cell Technologies”, Research Park, St. Petersburg State University (SPbSU), 199034 St. Petersburg, Russia; kirill.volkov@spbu.ru (K.V.V.); lankira@mail.ru (A.N.L.); alyx@bk.ru (A.N.I.); 6 Department of Biotechnology, All-Russia Research Institute for Agricultural Microbiology (ARRIAM), 196608 St. Petersburg, Russia; oshtark@yandex.ru (O.Y.S.); vzhukov@arriam.ru (V.A.Z.); 7 Komarov Botanical Institute RAS, 197376 St. Petersburg, Russia; 8 Peter the Great St. Petersburg Polytechnic University, 195251 St. Petersburg, Russia

**Keywords:** amyloid, fibril, RopA, RopB, outer membrane protein, porin, root nodule, bacteria, plant–microbial interaction, *Rhizobium leguminosarum*

## Abstract

Amyloids represent protein fibrils with a highly ordered spatial structure, which not only cause dozens of incurable human and animal diseases but also play vital biological roles in Archaea, Bacteria, and Eukarya. Despite the fact that association of bacterial amyloids with microbial pathogenesis and infectious diseases is well known, there is a lack of information concerning the amyloids of symbiotic bacteria. In this study, using the previously developed proteomic method for screening and identification of amyloids (PSIA), we identified amyloidogenic proteins in the proteome of the root nodule bacterium *Rhizobium leguminosarum*. Among 54 proteins identified, we selected two proteins, RopA and RopB, which are predicted to have β-barrel structure and are likely to be involved in the control of plant-microbial symbiosis. We demonstrated that the full-length RopA and RopB form bona fide amyloid fibrils in vitro. In particular, these fibrils are β-sheet-rich, bind Thioflavin T (ThT), exhibit green birefringence upon staining with Congo Red (CR), and resist treatment with ionic detergents and proteases. The heterologously expressed RopA and RopB intracellularly aggregate in yeast and assemble into amyloid fibrils at the surface of *Escherichia coli*. The capsules of the *R. leguminosarum* cells bind CR, exhibit green birefringence, and contain fibrils of RopA and RopB in vivo.

## 1. Introduction

Amyloids are fibrillar protein aggregates characterized by a highly ordered spatial structure called cross-β [1,2,3]. Amyloid fibrils are formed by intermolecular β-sheets stabilized by numerous hydrogen bonds that give rise to a cross-β X-ray diffraction pattern [2,3]. This type of structure determines amyloid stability, resulting in their resistance to treatment with protein denaturants like ionic detergents [4], as well as proteases [5]. Other specific properties of the amyloids include their binding with Thioflavin T (ThT) [6] and green or yellow birefringence in polarized light upon binding with Congo Red (CR) [7].

For more than 150 years, researchers drew attention to amyloid deposits due to their pathogenic roles. Until now, about 40 amyloid-forming proteins and peptides associated with the development of incurable human and animal diseases were discovered [1]. Nevertheless, the observations over the last decades demonstrated that amyloids can perform various physiological functions in prokaryotes and eukaryotes. For example, in fungi, amyloids are involved in the control of heterokaryon incompatibility [8], cell adhesion [9], spore formation and dispersal [10], and multicellularity [11]. Moreover, functional amyloids are found within animal species where they regulate long-term memory formation [12,13,14], peptide hormone storage [15], antiviral response [16], programmed necrosis [17], and melanin polymerization [18]. The majority of functional amyloids were identified in prokaryotes within both archaea and bacteria domains. In archaea, amyloids play important structural roles taking part in biofilm [19] or extracellular sheath formation [20]. Bacterial functional amyloids such as CsgA curlin from *Escherichia coli* [21] and Fap protein from *Pseudomonas* spp. [22] act as adhesins and components of biofilms [23]. Other functions of bacterial amyloids are lowering surface tension for aerial hyphae development in *Streptomyces coelicolor* [24,25] and toxin activity regulation in different species. For example, microcin E492 from *Klebsiella pneumoniae* [26] and listeriolysin O (LLO) from *Listeria monocytogenes* [27] are inactivated in the amyloid state, while amyloid harpins from *Xanthomonas axonopodis* are functionally active [28].

Most prokaryotic amyloids were found in species pathogenic for multicellular hosts but their occurrence in symbiotic species of bacteria was not analyzed. *Rhizobium leguminosarum* is a symbiotic nitrogen-fixing, root-nodule bacterium belonging to the order Rhizobiales, class Alphaproteobacteria. Such symbionts of legumes (Fabaceae) including species of the orders Rhizobiales and Burkholderiales (class Betaproteobacteria) form the paraphyletic group called “rhizobia” [29]. The ability of rhizobia to establish plant–bacteria symbiosis, resulting in their differentiation into large, motionless, non-dividing, and nitrogen-fixing cells called “bacteroids”, makes them not only economically and ecologically important objects of numerous studies, but also a widely studied model of complex plant–microbe interaction [30]. Since bacterial pathogenesis and symbiosis share common strategies such as host surface colonization, quorum sensing, and the use of type III and IV secretion systems for host response modulation [31,32], it seems plausible that amyloids might participate in the establishment of not only pathogenic but also mutualistic interactions. Therefore, in this work, we decided to identify potentially amyloidogenic proteins in the proteome of *Rhizobium leguminosarum* bv. *viciae* by using the previously developed proteomic screening and identification of amyloids (PSIA) approach [33], and we performed detailed analyses of their amyloid properties.

## 2. Materials and Methods 

### 2.1. PSIA Assay

For the identification of potentially amyloidogenic proteins that form polymers resistant to the treatment with ionic detergent sodium *N*-lauroylsarcosinate (sarcosyl, Helicon, Moscow, Russia) by PSIA, *Rhizobium leguminosarum* bv. *viciae* strain RCAM1026 [34] was used. *R. leguminosarum* culture was grown on a solid nutrient medium (0.5 g/L K_2_HPO_4_; 0.2 g/L MgSO_4_·7H_2_O; 0.1 g/L NaCl; 0.01 g/L CaCO_3_; 10 g/L mannitol; 0.4 g/L yeast extract; 15 g/L agar, Helicon, Moscow, Russia) supplemented by 500 µg/L streptomycin at 28 °C for 48 h. Cells (300 mg) were collected by a glass rod, suspended in 10 mL of phosphate-buffered saline (PBS, pH 7.4, Helicon, Moscow, Russia) with 3% sarcosyl, and subjected to PSIA.

The PSIA assay comprises four major steps: (i) the isolation and purification of proteins that form detergent-resistant polymers by using a series of ultracentrifugations of protein lysates with or without sarcosyl (final concentrations of sarcosyl in a sample of 3%, and 0.3% in sucrose cushion prior ultracentrifugation); (ii) the solubilization and trypsinolysis of the isolated protein polymers; (iii) the high-performance liquid chromatography (HPLC, Dionex UltiMate 3000 RSLCnano (Thermo Fisher Scientific, Waltham, MA, USA, chromatography system) of the obtained tryptic peptide mixtures; and (iv) the identification of proteins by MALDI-TOF/TOF (matrix-assisted laser desorption/ionization–time of flight/time of flight) mass-spectrometry using Ultraflextrime (Bruker Daltonics, Billerica, MA, USA). Carboxymethylation of cysteine, partial oxidation of methionine, and one skipped trypsinolysis site were considered as permissible modifications. “BioTools” program (Bruker Daltonics, USA) was used for manual validation of protein identification. A detailed description of PSIA was published previously [35] with modifications proposed for bacterial detergent-resistant protein extraction and purification [36].

### 2.2. Plasmids

For the analysis of amyloid properties of RopA and RopB proteins in the curli-dependent amyloid generator (C-DAG) system [37], pExport–RopA and pExport–RopB plasmids were constructed on the basis of pVS72 vector [37]. The codon optimization of RopA- and RopB-encoding sequences for expression in *E. coli* and cloning into pVS72 was performed by “Evrogen” company (Moscow, Russia) (File S1, Supplementary Materials). The pVS72-based plasmids for secretion of the control Sup35NM (amyloid) and Sup35M (soluble) proteins were obtained previously [37,38].

To analyze the aggregation of the RopA and RopB proteins fused with yellow fluorescent protein (YFP) in *Saccharomyces cerevisiae* cells, we constructed the pRS315–CUP1–RopA–YFP and pRS315–CUP1–RopB–YFP plasmids. The *ropA* and *ropB* genes were PCR-amplified by using FHindRopA and RBglRopA, and FHindRopB and RBamRopB pairs of primers (Table S1, Supplementary Materials), respectively, and the corresponding pExport-RopA or pExport-RopB pVS72-based plasmids as templates. The obtained fragments were cloned into the pRS315–CUP1–SIS1–YFP plasmid [39] by the *Hind*III and *Bam*HI restriction sites to substitute the *SIS1* sequence with either *ropA* or *ropB* fused in-frame with *YFP* under the control of the copper-inducible *CUP1* promoter. The p315L–Cup1–YFP plasmid that encodes YFP was constructed previously [40].

To construct pLATE–RopA and pLATE–RopB plasmids for the expression of RopA and RopB proteins fused with C-terminal 6×-His tag, *ropA* and *ropB* were PCR-amplified by using FAlicRopA and RAlicRopA, and FAlicRopB and RalicRopB pairs of primers (Table S1, Supplementary Materials), respectively, and corresponding pVS72-based plasmids as templates. The cloning of RopA- and RopB-encoding fragments into the pLATE vector was performed according to the manufacturer’s protocol (Thermo Fisher Scientific, Waltham, MA, USA). Correctness of the plasmids obtained was verified by sequencing with primers provided by the manufacturer (Thermo Fisher Scientific, Waltham, MA, USA).

### 2.3. Analysis of the YFP-Fused Protein Aggregation in Yeast Cells

The 1-OT56 strain (*MAT*a *ade1–14*_UGA_ *his3 leu2 trp1–289*_UAG_ *ura3* [*psi*^−^][*PIN*^+^]) strain [41] of *Saccharomyces cerevisiae* was transformed with pRopA–YFP or pRopB–YFP plasmids for target proteins production or p315L–Cup1–YFP plasmid that was used as a negative control. Transformants were chosen on selective medium (SC) [42], and then single colonies were inoculated in liquid selective medium supplemented with 150 μM copper sulfate to induce the *CUP1* promoter for overexpression of target proteins before growing for 6 h at 30 °C. The aggregate formation was analyzed by fluorescent microscopy using Axio Imager A2 transmitted light microscope (Carl Zeiss, Oberkochen, Germany) equipped with an enhanced contrast (EC) “Plan-Neofluar” 100×/1.3 Oil Ph3 objective and Colibri 7 fluorescent light-emitting diode (LED) light source (Carl Zeiss, Germany).

### 2.4. C-DAG Assay

The analysis of amyloid properties of RopA and RopB proteins was performed in a curli-dependent amyloid generator (C-DAG) system according to the previously published protocol [37] with modifications. Concisely, *E. coli* strain VS39 was transformed with pExport–RopA or pExport–RopB plasmids, and the selected transformants were placed on non-inducing LB (lysogeny broth, Miller: 10 g/L tryptone, 5 g/L yeast extract, 10 g/L NaCl, 20 g/L agar) plates supplemented with 100 μg/mL carbenicillin, 25 μg/mL chloramphenicol, and 0.5% glucose.

Single colonies were inoculated into liquid LB (100 μg/mL carbenicillin, 25 μg/mL chloramphenicol, Sigma-Aldrich, Saint-Louis, MO, USA) and incubated overnight at 37 °C. The overnight cultures were diluted with liquid LB (100 μg/mL carbenicillin, 25 μg/mL chloramphenicol) to optical density (OD) 0.01 and incubated for 30 min at 37 °C. Furthermore, 3 μL of the cultures were spotted onto the inducing LB plates (100 μg/mL carbenicillin, 25 μg/mL chloramphenicol, 0.2% l-arabinose, 1 mM isopropyl β-d-1-thiogalactopyranoside (IPTG), 20 μg/mL CR) and incubated at 30 °C for four days.

For the polarized light microscopy, *E. coli* cells exporting target proteins were suspended in PBS, placed onto the slide, and dried on air. The samples were stained with the water-based solution of CR (10 mg/mL), dried, and washed from the excessive dye with distilled water. Polarization microscopy was done by using Axio Imager A2 transmitted light microscope (Carl Zeiss, Germany) equipped with a Plan-Neofluar 40×/0.75 M27 objective (Carl Zeiss, Germany) and cross-polarizers.

### 2.5. Protein Production, Purification, and In Vitro Fibril Formation

The expression of RopA and RopB proteins was carried out in *E. coli* strain BL21 (DE3) (New England Biolabs, Ipswich, MA, USA) grown in 2TY liquid media (16 g/L tryptone, 10 g/L yeast extract, 5 g/L NaCl). We used 0.1 mM isopropyl β-d-1-thiogalactopyranoside (IPTG, Thermo Fisher, USA) to induce expression of proteins. After the induction of overproduction, cultures were grown for 4 h at 28 °C for RopA and 37 °C for RopB. The 6×-His-tagged proteins were purified in the presence of 8 M urea with the usage of Ni-NTA (nitrilotriacetic acid) agarose (Invitrogen, Carlsbad, CA, USA) column according to the protocol [43] without the Q Sepharose purification step. Purified proteins were concentrated with ethanol.

For the initiation of RopA and RopB aggregation in vitro, different buffers and incubation conditions were used: (i) the proteins in concentration 0.5 mg/mL were dissolved in 50% 1,1,1,3,3,3-hexafluoro-2-propanol (HFIP, Sigma-Aldrich, Saint-Louis, MO, USA) and incubated for seven days [44,45] Afterward, HFIP was evaporated under a stream of nitrogen, and the samples were stirred for an additional seven days. These conditions were also used for experiments with seeding. “Seeds” prepared on the basis of pre-incubated RopA and RopB aggregates were added to the samples at the beginning of fibrillogenesis in 1% (*v*/*v*) concentration; (ii) the proteins in concentration 0.5 mg/mL were incubated with constant stirring for seven days in 100 mM KH_2_PO_4_–NaOH in the presence of 3 M GdnHCl (pH 6.3) at 57 °C [46]; (iii) the proteins in concentration 0.5 mg/mL were incubated with constant stirring for seven days in 20% acetic acid solution in the presence of 100 mM NaCl (pH 2.0) at 37 °C [45,46]. For Aβ-peptide (1–42 amino acids (aa)) and lysozyme amyloid fibril preparation described above, conditions (i) and (iii), respectively, were used. Protein concentrations in experiments corresponded to those used in the case of RopA and RopB. Amyloid fibril formation was controlled by transmission electron microscopy (Libra 120, Carl Zeiss, Oberkochen, Germany).

### 2.6. Transmission Electron Microscopy (TEM)

Micrographs were obtained using the transmission electron microscope Libra 120 (Carl Zeiss, Oberkochen, Germany). The samples were placed on nickel grids coated with formvar films (Electron Microscopy Sciences, Hatfield, PA, USA). In order to obtain electron micrographs, the method of the negative staining with 1% aqueous solution of uranyl acetate (SPI Supplies, West Chester, PA, USA) was used.

Immunoelectron microscopy was performed as described in References [47] and [48] with modifications. We used 2.5% paraformaldehyde (Sigma-Aldrich, Saint-Louis, MO, USA)/PBS to fix the cells that were grown for 48 h on TY plates. The grids were blocked in 5% BSA (Amresco, Radnor, PA, USA)/PBST (supplemented with 1% Tween-20 (Helicon, Moscow, Russia)) for 15 min. Then, incubations with primary antibody (rabbit anti-RopA or anti-RopB (PrimeBioMed LLC, Moscow, Russia)) at 1:50 dilution (in blocking solution) for 24 h at 4 °C and secondary antibody conjugated with gold particle (goat anti-rabbit immunoglobulin G (IgG)–gold (Electron Microscopy Sciences, Hatfield, PA, USA)) at 1:40 (in blocking solution) for 45 min were performed. The carbon–formvar-coated copper (300 mesh) grids were washed thrice in blocking solution between incubations and finally in PBS (Helicon, Moscow, Russia) and ultrapure water. Grids incubated with gold-conjugated secondary antibody without the primary antibody were used as the negative control. In order to obtain electron micrographs, the method of the negative staining with 1% aqueous solution of uranyl acetate (SPI Supplies, West Chester, PA, USA) and Jeol JEM-1400 transmission electron microscope (JEOL, Akishima, Japan) were used.

### 2.7. ThT staining and Confocal Microscopy

ThT “UltraPure Grade” (AnaSpec, Fremont, CA, USA) without after-purification was used. ThT-fibril-tested solutions were prepared by equilibrium microdialysis using the Harvard Apparatus/Amika device (Harvard Apparatus, Holliston, MA, USA). Equilibrium microdialysis was performed with the concentration of aggregates ~0.5 mg/mL and initial concentration of ThT ~32 μM. The spectroscopic study of the sample and reference solutions prepared by proposed approach allowed us to determine the photophysical characteristics ThT bound to tested amyloids [45]. For obtaining fluorescence images of the ThT-stained fibrillar structures, we used the confocal laser scanning microscope Olympus FV 3000 (Olympus, Tokyo, Japan) equipped with the 60× oil immersion objective with a numerical aperture NA 1.42 and laser with excitation line 405 nm.

### 2.8. Absorbtion and Fluorescence Measurements

The absorption spectra of the samples were recorded using a U-3900H spectrophotometer (Hitachi, Tokyo, Japan). The turbidity of the samples containing fibrils was monitored by the measuring absorbance at 350 nm. Fluorescence and fluorescence excitation spectra were measured using a Cary Eclipse spectrofluorometer (Varian, Palo Alto, CA, USA). For Rayleigh light scattering determination, samples with amyloid aggregates were excited at 350 nm and registered at 350 nm. Fluorescence of ThT was excited at a wavelength of 440 nm and registered at 490 nm. A PBS solution of ATTO-425, whose fluorescence and absorption spectra are similar to that of ThT, was taken as a reference for determining the fluorescence quantum yield of ThT bound to fibrils. The fluorescence quantum yield of ATTO-425 was taken as 0.9 (ATTO-TEC Catalogue 2009/2010 p.14). The spectral slit width was 5 nm in the majority of experiments. Changing the slit widths did not influence the experimental results. Circular dichroism (CD) spectra in the far-ultraviolet (UV) region were measured using a J-810 spectropolarimeter (Jasco, Tokyo, Japan). Spectra were recorded in a 0.1 cm cell from 260 to 198 nm. For all spectra, an average of three scans was obtained. The CD spectrum of the appropriate buffer was recorded and subtracted from the sample spectra. Fluorescence decay curves were recorded by a spectrometer FluoTime 300 (PicoQuant, Berlin, Germany) with the Laser Diode Head LDH-C-440 (λ_ex_ = 440 nm). The fluorescence of ThT was registered at λ_ex_ = 490 nm.

### 2.9. CR Staining and Polarization Microscopy of Fibrils

Protein samples (3 µL) were placed on microscope slides and air-dried. Then the samples were stained with 40 µL of water-based saturated CR dye solution (10 mg/mL, filtered through a 0.45 µL filter (Merck Millipore, Burlington, MA, USA)) and air-dried. The excessive dye was removed by washing slides twice with distilled water. The analysis of birefringence was performed using an Axio Imager A2 transmitted light microscope (Carl Zeiss, Oberkochen, Germany) equipped with cross-polarizers and a Plan-Neofluar 40×/0.75 M27 objective (Carl Zeiss, Oberkochen, Germany).

### 2.10. Analysis of Detergent and Protease Resistance of Protein Aggregates and Immunodetection

SDS–PAGE (sodium dodecyl sulfate–polyacrylamide gel electrophoresis) was performed according to the standard protocol [49]. Before loading on the SDS–PAGE gel, Laemli SDS–PAGE sample buffer containing SDS (final concentration 2%, Bio-Rad, Hercules, CA, USA) was added, and samples were incubated at room temperature or boiled (as indicated in the text) for 5 min. For the experiments with trypsin or pepsin digestion, the samples of the in vitro obtained RopA and RopB proteins (1 mg/mL) were treated with trypsin or pepsin (Sigma-Aldrich, Saint-Louis, MO, USA) at 1:60 to total protein mass ratio for 30 min at 37 °C. The in vitro obtained *S. cerevisiae* Sup35NM protein sample was used as the control.

For experiments with *R. leguminosarum*, cells were grown in a liquid tryptone–yeast medium (5 g/L tryptone, 3 g/L yeast extract, and 1.3 g/L CaCl_2_·6H_2_O) at 28 °C with shaking. Overnight culture was diluted 1/25 into tryptone–yeast medium supplemented (if required) with 10 mM luteolin (Sigma-Aldrich, Saint-Louis, MO, USA) and grown for 6 h under the same conditions. Cells were collected by centrifugation at 3500× *g* for 30 min at 4 °C, and the pellet was suspended in PBS supplemented with 0.5 mM phenylmethylsulfonyl fluoride (Sigma-Aldrich, Saint-Louis, MO, USA). To obtain total protein lysates, treatment with 2% SDS and 0.5% Tween-20 at room temperature for 5 min followed by pulse sonication (Q125, Qsonica, Newtown, CT, USA) at 40% power for 10 s was used.

After the incubation, protein samples were incubated with SDS-PAGE sample buffer (containing SDS, final concentration 2%) with or without boiling at 100 °C for 5 min prior the loading onto the gel and analyzed by Western blot. Mini Trans-Blot Cell system (Bio-Rad, Hercules, CA, USA) was used to perform wet transfer from SDS–PAGE gel onto a polyvinylidene fluoride (PVDF) membrane (Amersham, Buckinghamshire, UK) followed by Western blot hybridization [49] for protein immunodetection. The SDD–AGE (semi-denaturing detergent–agarose gel electrophoresis) assay was performed as described previously [50] with capillary transfer modification [51].

For Western blot analysis of the in vitro obtained proteins, mouse monoclonal 6×-His antibody 4A12E4 (Invitrogen, USA) was diluted 1:5000 to detect RopA and RopB proteins fused with a 6×-His tag. A goat anti-mouse IgG (H + L) secondary antibody (Abcam, Cambridge, UK) was also used in dilution 1:5000 to detect the 6×-His tagged proteins.

To detect intact RopA and RopB proteins extracted from *R. leguminosarum* cells, rabbit anti-RopA and anti-RopB antibodies (PrimeBioMed LLC, Moscow, Russia) and secondary goat anti-rabbit IgG (H + L) antibody (Thermo Scientific, Waltham, MA, USA) were used in the dilutions 1:1000 and 1:20,000, respectively. Visualization of protein signals by enhanced chemiluminescence (ECL) Prime Western Blotting Detection reagent (GE Healthcare, Chicago, IL, USA), and Bio-Rad ChemiDoc™ hardware and software (Bio-Rad, Hercules, CA, USA) was used for imaging.

### 2.11. Data Analysis

The experiments were performed with at least three repeats. Data in figures are presented as the means ± the standard errors of the means (SEM).

The absorption spectra of proteins aggregates and ThT in their presence were analyzed along with the light scattering using a standard procedure [52]. The concentrations of RopA, RopB, and ThT in solutions were determined using the molar extinction coefficients of ε_280_ = 38,050 M^−1^∙cm^−1^, ε_280_ = 21,588 M^−1^∙cm^−1^, and ε_412_ = 31,589 M^−1^∙cm^−1^, respectively. Recorded fluorescence intensity was corrected on the primary inner filter effect with the use of previously elaborated approach [53]. The quantitative analysis of the secondary structure was carried out by the CDPro program using three different regression methods (Selcon, Contin, and CDSSTR) and several basic sets of proteins with a known secondary structure (the sets include from 37 to 56 soluble, membrane, and denatured proteins with different secondary structure content).

The measured emission decays were fit to a multiexponential function using the standard convolute-and-compare nonlinear least-squares procedure [54]. The fitting routine was based on the nonlinear least-squares method. Minimization was performed according to Marquardt [55].

## 3. Results

### 3.1. Proteomic Screening Revealed Potentially Amyloidogenic Proteins of R. leguminosarum

Initially, we decided to identify potentially amyloidogenic proteins in the proteome of *R. leguminosarum* using the previously developed PSIA approach [35,36]. That method is based on an unusual property of amyloids, i.e., their resistance to treatment with ionic detergents [4]. To identify proteins of *R. leguminosarum* that form detergent-resistant polymers and complexes in vivo by PSIA, we isolated and purified protein fractions resistant to treatment with ionic detergent sarcosyl (3%), solubilized and trypsinized them, separated tryptic peptides by HPLC, and identified proteins by MALDI TOF/TOF mass spectrometry.

**Figure 1:**
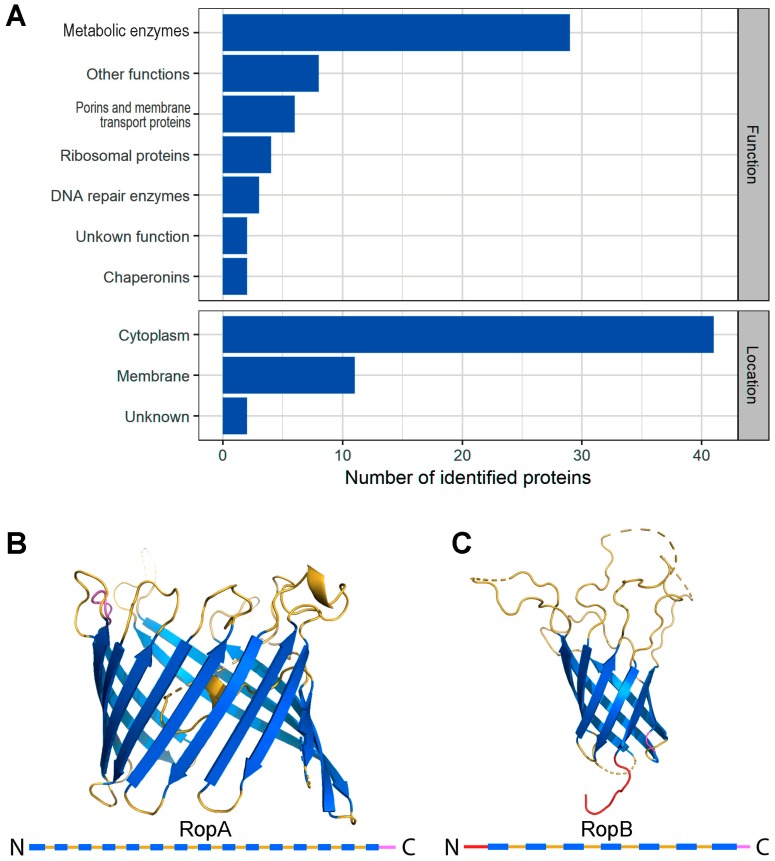
Detergent-resistant fractions of *Rhizobium leguminosarum* and proteins comprising them. (**A**) Functions and locations of proteins comprising detergent-resistant fractions based on UniProt data (https://www.uniprot.org/). (**B**,**C**) Structures of RopA and RopB proteins, respectively, predicted with I-TASSER server [56]. Secondary structures of RopA and RopB are presented below with bars. Blue bars denote beta-sheets, yellow ones denote loops, and red and purple bars denote the *N*-terminus and *C*-terminus, respectively.

The results of identification demonstrated the presence of 54 proteins in detergent-resistant fractions of the *R. leguminosarum* proteome (Table S2, Supplementary Materials). Based on data of UniProt, the majority of these proteins are cytoplasmically located and functionally related to metabolic enzymes, while membrane proteins comprise about one-fifth of their number (Figure 1A) The identified proteins were predicted to act as porins and transporters, ribosomal proteins, DNA repair enzymes, and chaperonins (Figure 1A). Within detergent-resistant proteins, RopA (Om3A) protein was identified with the highest mass spectrometric score and contained typical features for the outer membrane protein β-sheet-rich barrel domain (Figure 1B, Figure S1, Supplementary Materials) suggesting its potentially amyloidogenic properties. The second probable outer membrane protein identified in our screening, RopB, had a similar barrel domain (Figure 1C). Based on the high propensity to form β-sheets, we selected RopA and RopB for a detailed analysis of their ability to form amyloids.

### 3.2. Full-Length RopA and RopB Form Fibrils In Vitro

**Figure 2:**
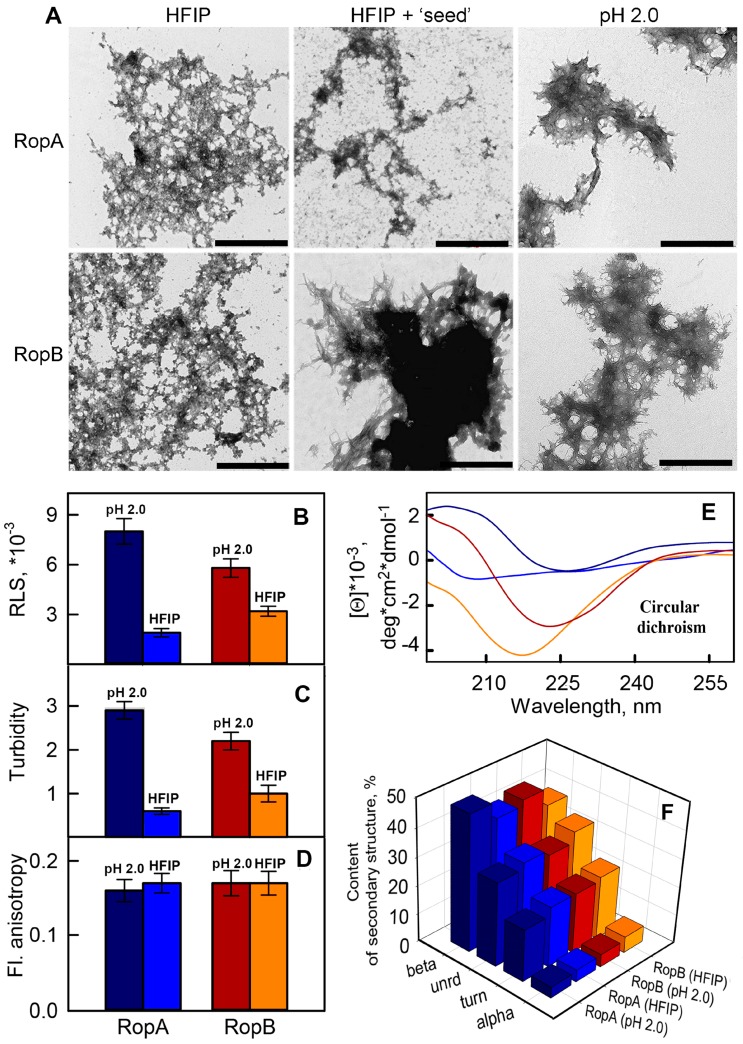
RopA and RopB form morphologically and structurally distinct aggregates in different conditions. (**A**) TEM images of RopA (top panels) and RopB (bottom panels) aggregates, obtained in various conditions: using an organic solvent 1,1,1,3,3,3-hexafluoro-2-propanol (HFIP) without (left panels) and with (middle panels) “seeding” and using a buffer with acidic pH 2.0 (right panels). The scale bar on the electron microscopy images is equal to 1 μm. (**B**) Rayleigh light scattering (RLS), (**C**) turbidity, (**D**) fluorescence anisotropy, and (**E**) circular dichroism (CD) of amyloids. (**F**) Content of elements of the secondary structure in aggregates: beta-sheets (beta), unordered structure (unrd), turns (turn), and alpha-helices (alpha). Spectra and bars colors of the panels (**B**–**F)** denote characteristics of the RopA aggregates prepared at pH 2.0 (dark-blue color) or using HFIP without “seeding” (blue) and RopB aggregates prepared at pH 2.0 (dark-red color) and using HFIP without “seeding” (orange).

At the first step of RopA and RopB amyloid properties analysis, it was important to understand whether these proteins form fibrillar aggregates. For this purpose, we expressed full-length recombinant RopA and RopB in *E. coli*, extracted and purified them, and tried to dissolve them in phosphate buffer (pH 7.4) at the room temperature. However, it turned out that proteins were poorly soluble and had a tendency to precipitate in these conditions, which did not allow inducing amyloidogenesis. To solve this problem and increase the solubility of the proteins, we used the buffer containing an organic solvent HFIP (see Section 2) which was previously used in other works to obtain amyloid fibrils [44,45]. The obtained TEM data showed that, after HFIP removing from the sample, both RopA and RopB formed mostly unstructured aggregates with the admixture of more ordered fibril-like structures (Figure 2A, left images).

Amyloid formation might often be initiated by using the “seeds”, prepared on the basis of pre-existing fibrils [57]. We tried to shift the equilibrium toward the formation of fibrils from RopA and RopB using the “seeds”, prepared on the basis of pre-incubated RopA and RopB aggregates (while maintaining the conditions of fibrillogenesis). Nevertheless, only RopB in the presence of the seeds formed long thin fibrillary structures prone to interacting with each other and clusterization (Figure 2A, central images). At the same time, RopA aggregates obtained using the “seed” did not differ in their morphology from those prepared in the absence of “seed” (Figure 2A, left images). In this regard, we decided to test whether there were other conditions of aggregation in which both proteins would be mostly fibrillar.

To obtain amyloid fibrils in vitro, the effects denaturing the structure of monomeric proteins are often used. These effects lead to the transition of the protein to a partially folded state and exposing the amyloidogenic fragments of the amino-acid sequence to a protein surface with the subsequent interaction of these fragments with each other and the formation of ordered aggregates. We tried to use denaturing effects such as the presence of a denaturation agent (GdnHCl), extremely high temperature, and extremely low pH. The latter conditions, indeed, allowed us to solve the problem successfully. For fibrillogenesis, we used a buffer solution consisting of 20% acetic acid in the presence of 100 mM NaCl (pH 2.0). These conditions were proposed earlier for the preparation of alpha-lactalbumin amyloid fibrils [58] and were successfully applied by us to obtain insulin and lysozyme amyloid fibrils [45,46]. RopA and RopB proteins were incubated in this solution for seven days at 37 °C with constant stirring. Using TEM, we showed that both obtained samples are very thin fibrous aggregates with a high tendency to clustering (Figure 2A, right images). In fact, we observed large clots fringed by fine fibers. Our next task was a comparative study of the structure and properties of the different types of aggregates and analysis of their amyloid properties. For a correct comparison of the prepared samples, they were investigated under the same conditions, for which the aggregates obtained in the acidic solution were preliminarily transferred to distilled water. The preservation of aggregate structure after this transfer and their stability under new conditions were controlled.

### 3.3. In Vitro Obtained RopA and RopB Fibrils Exhibit Amyloid Properties

#### 3.3.1. Photophysical Properties of the RopA and RopB Fibrils and Aggregates

For a comparative study of the prepared aggregates, we firstly analyzed their photophysical properties. It turned out that Rayleigh light scattering (RLS) and turbidity of aggregates formed from the same protein under two different conditions (in the presence HFIP and in acidic buffer solution) differed significantly (Figure 2B,C). This confirmed the differences in sizes of the studied aggregates observed by TEM (Figure 2A, left and right panels). Large clots of fibrils formed in acidic conditions scattered light more efficiently in comparison to aggregates formed in the presence of HFIP. At the same time, values of the fluorescence anisotropy of the samples were close, which indicated a similar intramolecular mobility of tryptophan residues in aggregated proteins (Figure 2D). It means that tryptophan residues have a similar microenvironment that, in particular, may be due to the fact that protein fragments in which tryptophan residues are localized are involved in the formation of similar elements of secondary structure.

For analyzing the secondary structure of obtained RopA and RopB aggregates, their circular dichroism (CD) spectra in the far-UV spectral region were registered. First of all, we made a comparison of the CD spectra of monomeric proteins in HFIP/water mixture and fibrils obtained in the presence of this solvent (Figure S2A, Supplementary Materials). It turned out that the content of the β-structure in both proteins was quite high (more than 40% and 30% for RopA and RopB, respectively, Figure S2B,C, Supplementary Materials), which was consistent with the content of the β-sheets in the β-barrel proteins. Considering these results, as well as the fact that, after dissolution of the RopA and RopB, HFIP was removed from the solutions, we can assume that these conditions did not have a significant denaturing effect on these proteins and may be close to physiological. Unfortunately, acidic buffer (pH 2.0) did not allow us recording the CD spectra of monomeric proteins in other conditions. However, due to the transfer of mature aggregates formed by RopA and RopB in these conditions into water, we were able to register their CD spectra.

The shape of the spectra and their value of CD are significantly different, indicating the structural polymorphism of the RopA and RopB aggregates prepared under different conditions (Figure 2E). We estimated the content of various types of the samples secondary structure by the CDPro program using three different regression methods and several basic sets of proteins with a known secondary structure that included from 37 to 56 soluble, membrane, and denatured proteins. It was shown that the content of beta-sheets which take part in the formation of the core of fibrillar amyloid aggregates in the samples prepared in solution with acidic pH was higher (about 48% and 44% for RopA and RopB aggregates, respectively) than that of the samples prepared using HFIP (about 42% and 38% for RopA and RopB aggregates, respectively) (Figure 2F). Despite the indicated differences, the content of the beta-structure in each sample was rather high, which is typical for amyloids. At the same time it can be noted that the light scattering of macromolecules can substantially distort the CD spectra (see, for example, Reference [59]), and this distortion may be different for different samples. In particular, some of the recorded CD spectra had an evident distortion in the region of 250–260 nm (difference of recorded values from 0) which was determined by the large size of the studied aggregates (Figure 2A). In addition, the standard basic sets of proteins used to estimate the secondary structure content were not representative of the analysis of the spectra of protein aggregates. Thus, despite the fact that the results obtained by CD spectroscopy did not contradict the assumptions about the amyloid nature of the prepared aggregates, we further investigated the samples by approaches that were more specific to amyloid fibrils.

#### 3.3.2. Fibrils of RopA and RopB Bind ThT

**Figure 3:**
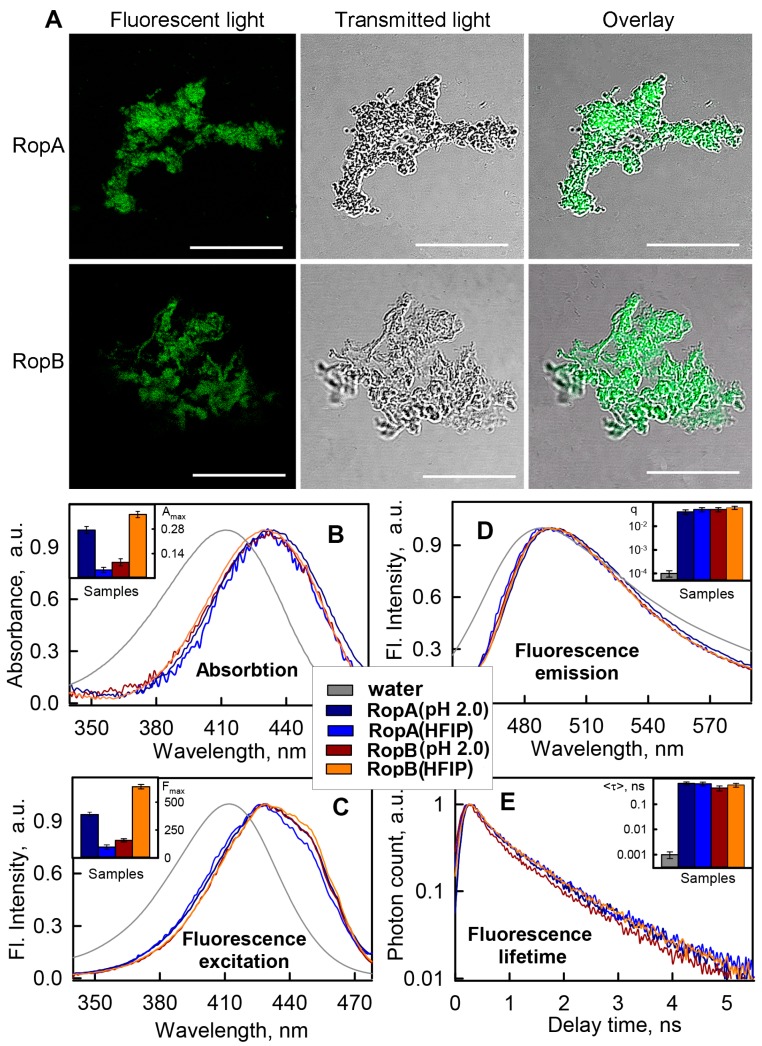
RopA and RopB aggregates binding with amyloid specific probe thioflavin T (ThT). (**A**) Confocal microscopy of prepared using HFIP aggregates stained with ThT. Fluorescence images of the ThT-stained aggregates structures (left panels), transmitted light images showing the presence of aggregates in investigated areas of the sample (middle panels), and overlay of the images (right panels) are presented. The scale bar on the images is equal to 20 μm. Normalized to unity at the maximum (**B**) absorption, (**C**) fluorescence excitation, (**D**) emission spectra, and (**E**) fluorescence decay curves of the ThT bound to amyloid fibrils. The insets of the corresponding panels show the maximum values of the absorption and fluorescence excitation, and the values of the fluorescence quantum yield and lifetime of the bound to fibrils dye. The decoding of the colors used is shown in the figure.

To check whether the tested aggregates were fibrillar, we examined their interaction with Thioflavin T (ThT), representing one of the most commonly used dyes for amyloid staining [6,60,61]. A unique feature of this dye is a change in its photophysical characteristics in the presence of amyloid fibrils (first of all, a significant increase in the dye fluorescence intensity) [62] and high specificity of ThT–fibril interaction. Therefore, we firstly checked whether the dye was able to bind to the aggregates that were obtained in the presence of HFIP which did not show fibrillar structure using TEM. It turned out that both RopA and RopB aggregates were effectively stained by ThT (Figure 3A), indicating the presence of fibrillar structures in these samples. However, due to the low resolution of confocal spectroscopy, it was impossible to visualize the fibrillary areas in these aggregates. Additional evidence for the presence of fibrillar structures in the tested samples could be obtained by using spectroscopic study of aggregates in the presence of ThT.

First of all, we recorded ThT fluorescence spectra in the presence of monomeric RopA and RopB and their aggregates in various conditions (Figure S3, Supplementary Materials). As expected, despite the high content of β-structures, a significant increase in the total fluorescence intensity of the dye in the presence of monomeric proteins did not occur. This fact confirmed the specificity of ThT binding to amyloid fibrils. As a positive control, we presented data for model amyloid fibrils formed from lysozyme and Aβ-peptide (1–42 aa) in the same buffers that were used for RopA and RopB fibrillogenesis. It turned out that an increase in the dye fluorescence intensity upon binding to lysozyme and Aβ amyloid fibrils was comparable (in order of magnitude) with an increase in its fluorescence intensity upon interaction with RopA and RopB aggregates obtained under various conditions, confirming their amyloidogenic nature.

According to modern concepts, the specificity of ThT interaction with amyloid fibrils is due to the fact that the dye binding sites are located in the grooves formed by side chains of amino-acid residues (that form the core of amyloid fibrils), along the long axis of the fibrils perpendicular to the beta-sheets [63]. Therefore, relying on the analysis of the photophysical characteristics of ThT bound to amyloids, we can identify not only the presence of amyloid fibrils in a sample but also the features of their structure. However, the latter task is complicated by the fact that a sample of ThT with fibrils is always in an equilibrium system of free dye and dye bound to fibrils (wherein the proportion of free dye is often very high), and the separation of the characteristics of different dye fractions for a long time was a nontrivial problem. To solve it, we previously proposed a special approach, based on the preparation of tested solutions using an equilibrium microdialysis [45,46]. This approach allowed us to obtain a sample solution containing amyloid fibrils and two equilibrium ThT fractions (free and bound dye), as well as a reference solution containing only free dye at a concentration equal to the concentration of free dye in the sample solution.

Using solutions prepared by the equilibrium microdialysis, we determined absorption, fluorescence emission and fluorescence excitation spectra, and fluorescence decay curves of ThT bound to RopA and RopB aggregates prepared in different conditions (Figure 3B–E). Spectral properties of the dye molecules bound to different types of fibrils turned out to be similar. The longer wavelength position of the absorption and excitation spectra of bound ThT molecules in comparison to that of free dye (Figure 3B,C) could have been caused by its more hydrophobic microenvironment into the fibrils grooves. It should be noted that, in the fluorescence excitation spectra of bound ThT, along with a maximum at a wavelength of about 430 nm, there was an even longer wavelength shoulder (about 450 nm), indicating the existence of two different types (modes) of the dye binding. A binding mode characterized by a maximum of the absorption and fluorescence excitation spectra at about 450 nm was previously detected when ThT bound to model amyloid fibrils formed from insulin and lysozyme [45,46]. On the basis of analysis of the ThT interaction with amyloid fibrils formed from a wide range of proteins and peptides, we proposed in our works that the second type of dye binding occurs during the clumping of amyloids (which according to TEM data is also a characteristic of RopA and RopB fibrils). In this case, the bound dye is located in an even more rigid and hydrophobic microenvironment compared to the first binding type, which leads to a longer wavelength shift in the absorption and fluorescence excitation spectra of the bound dye.

Analysis of the shape and position of the recorded ThT fluorescence spectra allowed us to conclude that fluorescence correction for the secondary inner effect was not required. The maximum of fluorescence spectra of the bound ThT coincided with the maximum of that of the free dye, as in the case of ThT interaction with amyloid fibrils formed from other amyloidogenic proteins [64]. The narrower fluorescence spectrum of the bound dye can be explained by the fewer possible conformations of ThT in the bound state due to the rigidity of the microenvironment and, consequently, to limiting of the torsion angle between dye fragments. In addition, the rigidity of the microenvironment led to a significant increase in the fluorescence quantum yield and lifetime of the bound dye (Figure 3D,E, insets), which is also a characteristic feature of the ThT interaction with amyloid fibrils.

The observed similarity of the photophysical parameters of ThT bound to RopA and RopB aggregates obtained under various conditions indicated the formation of amyloid fibrils with similar structure. At the same time, it was found that the absorption (concentration) and, hence, the fluorescence intensity of the dye bound to the fibrils differed significantly for diverse fibril types (Figure 3B,C, insets), which may indicate their different amounts in the samples. This fact was also consistent with the data of experiments that showed differences in the secondary structure of the fibrils (Figure 2E,F). Thus, the obtained data demonstrate that RopA and RopB form fibrils in vitro, and the propensity of proteins to fibrillogenesis depends on the conditions inducing this process. At the same time, it was also possible to assume the simultaneous formation of RopA and RopB aggregates with a non-fibrillar structure in some conditions, whereby they were adsorbed onto the surface of fibrils and did not allow identifying the fibril structure by TEM.

#### 3.3.3. Fibrils of RopA and RopB Exhibit Green Birefringence upon Binding with CR

**Figure 4:**
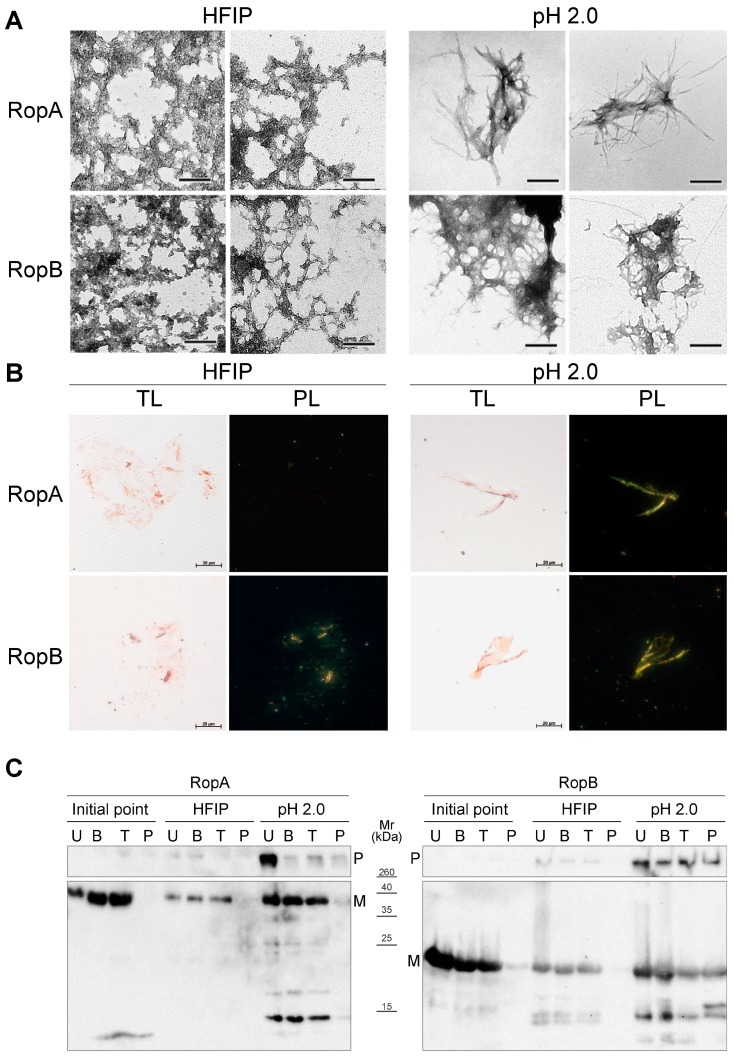
Amyloid properties of RopA and RopB fibrils formed in vitro. (**A**) TEM images of the in vitro obtained RopA and RopB aggregates and fibrils prepared either in HFIP or acidic conditions (pH 2.0). Scale bar is equal to 200 nm. (**B**) Polarized light microscopy of the Congo Red (CR)-stained RopA and RopB samples prepared either in HFIP or acidic conditions (pH 2.0). TL—transmitted light, PL—polarized light. Scale bar is equal to 20 μm. (**C**) Detergent and protease resistance of the RopA and RopB aggregates and fibrils detected by Western blot. Initial point—proteins solubilized in phosphate-buffered saline (PBS); HFIP and pH 2.0—protein aggregates were obtained in the corresponding buffers. U—unboiled samples treated with 2% cold SDS; B—boiled with 2% SDS; T—treated with trypsin; P—treated with pepsin; P—polymers; M—monomers. Corresponding molecular weights (kDA) are indicated.

To analyze the interaction of RopA and RopB aggregates with CR, we used samples prepared either in HFIP where both proteins formed less ordered aggregates or in buffer solution with pH 2.0 where fibril formation prevailed (Figure 4A). In vitro obtained amyloid aggregates formed by the Sup35NM fragment of the *S. cerevisiae* release factor, as well as the Sup35M soluble fragment of the same protein, were used as the positive and negative controls, respectively (Figure S4, Supplementary Materials). Binding of amyloids with CR leads to green or yellow birefringence in polarized light [1], indicating presence of a cross-β structure typical for amyloid fibrils [2,3]. All samples analyzed bound CR, resulting in their red color in transmitted light (Figure 4B, transmitted light (TL) panels). Staining of the RopA and RopB fibrils obtained at pH 2.0 with CR revealed green birefringence under polarized light, indicating their amyloid structure (Figure 4B, polarized light (PL) panels). At the same time, staining of the RopA and RopB samples obtained in HFIP caused birefringence in the polarized light only in the RopB sample, while RopA did not exhibit birefringence (Figure 4B, PL panels). This suggests that either RopA fibrils obtained in HFIP were less ordered, or unstructured aggregates presented in this sample masked surfaces of the fibrils and titrated CR, thus preventing CR binding with amyloids. Thus, despite RopA and RopB aggregating in HFIP and at pH 2.0, only the latter conditions allowed obtaining bona fide amyloid fibrils of both these proteins, indicating that acidic pH may be more favorable for amyloid formation by the hydrophobic membrane proteins.

#### 3.3.4. Detergent and Protease Resistance of Amyloids

One of the characteristic properties of amyloids is their high resistance to treatment with ionic detergents and proteases [4,5]. To analyze the resistance of the RopA and RopB aggregates to treatment with sodium dodecyl sulfate (SDS) ionic detergent, we incubated RopA and RopB samples obtained in different conditions (Figure 4C) in buffer containing 2% SDS for 5 min at room temperature or upon boiling them at 100 °C, and we subjected the samples to Western blot analysis. Results of this experiment demonstrated that RopA and RopB samples obtained immediately after solubilization did not form detergent-resistant aggregates (Figure 4C). Samples of RopA and RopB prepared in HFIP formed weak bands of detergent-resistant aggregates that stayed in the wells without entering the gel (Figure 4C). This was consistent with their composition presented by unstructured aggregates with an admixture of fibrils (Figure 4A). Finally, RopA and RopB fibrils prepared at pH 2.0 formed robust bands of detergent-resistant aggregates, but while RopA fibrils were resistant to only cold SDS, fibrils of RopB exhibited high resistance to both cold and hot SDS (Figure 4C).

We analyzed resistance of RopA and RopB to proteolytic digestion. For this experiment, the same samples as for analysis of detergent resistance were used. These samples were treated with either trypsin or pepsin proteases, boiled in SDS–PAGE sample buffer, loaded onto the gel, and subjected to Western blot. As a result, differences in stability of RopA and RopB were also revealed (Figure 4C). While both RopA and RopB exhibited resistance to treatment with trypsin (Figure 4C), only RopB fibrils prepared at pH 2.0 resisted treatment with pepsin, demonstrating their high stability to proteolytic digestion (Figure 4C). The control Sup35NM sample (in the non-amyloid state obtained immediately after solubilization) was neither resistant to trypsin nor pepsin treatment (Figure S5, Supplementary Materials). Overall, a series of the aforementioned experiments demonstrated that the in vitro obtained fibrils of RopA and RopB exhibited all key characteristics of bona fide amyloids.

### 3.4. RopA and RopB Demonstrate Amyloid Properties In Vivo

**Figure 5:**
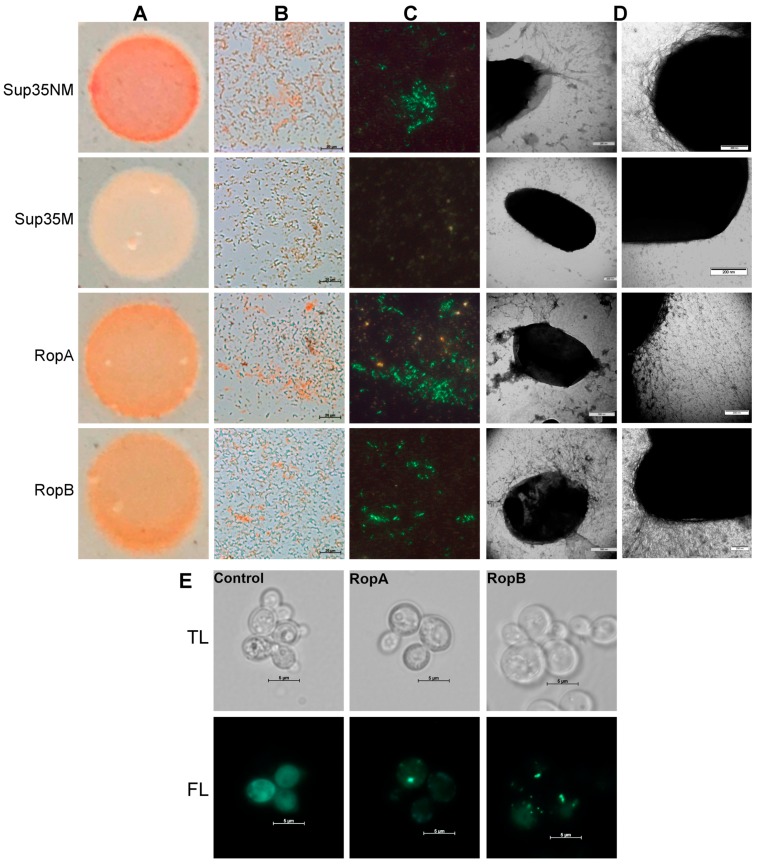
Analysis of the RopA and RopB amyloid properties in the *Escherichia coli* and *Saccharomyces cerevisiae* cells. (**A**–**D**) RopA and RopB extracellular secretion in the *E. coli* curli-dependent amyloid generator (C-DAG) system. (**A**) Bacterial colonies on the media containing CR. (**B**) CR-stained bacterial cells in transmitted and (**C**) polarized light. Scale bar is equal to 20 μm. The *E. coli* cells secreting Sup35NM (amyloid) and Sup35M (soluble) protein fragments were used as the positive and negative controls, respectively. (**D**) TEM images of *E. coli* exporting target proteins. Different magnifications are shown. (**E**) Analysis of aggregation of RopA and RopB proteins fused with yellow fluorescent protein (YFP) in the cells of yeast *S. cerevisiae*; TL—transmitted light, FL—fluorescent light. The YFP protein was used as a negative control. Scale bar is equal to 5 μm.

To analyze the ability of RopA and RopB proteins to aggregate in living cells, we used an *Escherichia coli*-based C-DAG (curli-dependent amyloid generator) system, which enables secretion of target proteins fused with the CsgA curlin signal sequence through a CsgG-formed pore to the surface of *E. coli* cell [37]. Secretions of the Sup35NM amyloid-forming and Sup35M soluble protein fragments were used as positive and negative controls, respectively. Initially, we analyzed the color of colonies secreting RopA, RopB, and control proteins on the plates with media containing CR. Cells secreting RopA and RopB exhibited a bright-orange color, indicating binding of CR (Figure 5A). The polarization microscopy study demonstrated green birefringence in cells expressing RopA and RopB, similar to those formed by the control amyloid protein Sup35NM (Figure 5B,C). Finally, the TEM study indicated the formation of the RopA and RopB fibrils at the surface of *E. coli* cells (Figure 5D).

To test whether RopA and RopB proteins are prone to intercellular aggregation, we constructed pRopA–YFP and pRopB–YFP plasmids that encode target proteins fused with yellow fluorescent protein (YFP) under the control of the copper-inducible *CUP1* promoter. Yeast *S. cerevisiae* were transformed by these plasmids, and fluorescence was analyzed after induction in the liquid copper-containing selective media. Results of fluorescence microscopy demonstrated that both RopA and RopB formed fluorescent aggregates in *S. cerevisiae* cells (Figure 5E). The aggregates of both proteins had morphology of multiple dots and formed after 6 h of induction. Thus, RopA and RopB formed amyloid fibrils at the surface of *E. coli* and aggregated in *S. cerevisiae.*

**Figure 6:**
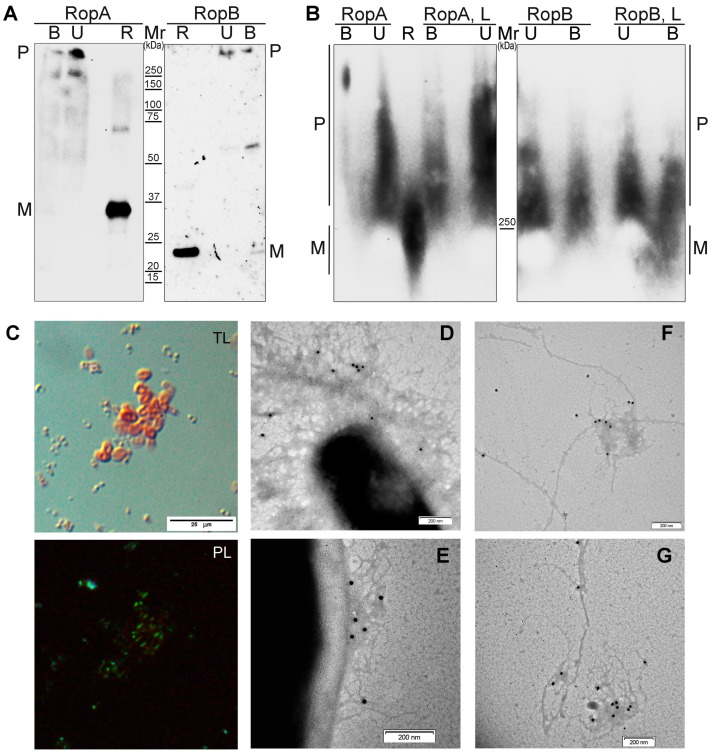
Amyloid properties of RopA and RopB in the *R. leguminosarum* cells. (**A**,**B**) Detection of the RopA and RopB detergent-resistant aggregate formation in *R. leguminosarum* by sodium dodecyl sulfate–polyacrylamide gel electrophoresis (SDS–PAGE) (**A**) and semi-denaturing detergent–agarose gel electrophoresis (SDD–AGE) (**B**). U—unboiled samples treated with cold 2% SDS; B—samples were boiled in the buffer containing 2% SDS; L—cells were grown in the presence of 10 mM luteolin, R—control, recombinant monomeric proteins RopA or RopB, respectively. Corresponding molecular weights (kDA) are indicated. (**C**) Transmitted light (TL) and polarized light (PL) microscopy of the *R. leguminosarum* cells incubated for seven days on TY plates. (**D**–**G**) TEM image of the *R. leguminosarum* cells and extracellular material labeled with either anti-RopA (**D**,**F**) or anti-RopB (**E**,**G**) antibodies visualized by gold-conjugated secondary antibodies. Scale bars are indicated in images.

To analyze formation of the RopA and RopB aggregates in vivo, we extracted protein lysates from the *R. leguminosarum* cells and analyzed these proteins by SDS–PAGE and SDD–AGE, followed by Western blot with the anti-RopA and anti-RopB antibodies, respectively. Since resistance to treatment with ionic detergents represents one of the key amyloid features, we treated total protein lysates of *R. leguminosarum* cells with 2% SDS and, additionally, with 0.5% polysorbate non-ionic surfactant Tween-20, incubated for 5 min at room temperature, and loaded onto the gels either without boiling or with boiling at 100 °C for 5 min. Results of SDS–PAGE analysis demonstrated that both RopA and RopB formed detergent-resistant polymers. While RopB polymers were partially dissolved to monomers, RopA did not form monomers even after boiling in the presence of 2% SDS (Figure 6A). The SDD–AGE analysis demonstrated that both proteins formed large polymers that entered the agarose gel and that were resistant to detergent treatment. The addition of the flavonoid compound luteolin that enhances expression of several virulence-related genes including *nod* and *ropA* [65,66] to the cultural media resulted in an increase in the amount and size of the RopA aggregates but did not affect RopB (Figure 6B).

Finally, to analyze the presence of amyloids in the *R. leguminosarum* cells in vivo, we stained them with CR. We found that *R. leguminosarum* cells were highly congophylic, and CR signals formed bright-red rings surrounding the cells (Figure 6C). These ring-like structures corresponded to capsules, specific extracellular structures predominantly composed of polysaccharides that are known to be produced by *R. leguminosarum* [67]. The ability of *R. leguminosarum* to bind CR was increased during incubation on the plates with TY media (seven-day culture was more congophylic than the one-day culture) that corresponded to the formation of capsules that occurred during the stationary growth phase [67]. Polarization microscopy detected strong green birefringence, indicating the presence of amyloid fibrils in the capsules (Figure 6C). Immunoelectron microscopy with gold-conjugated antibody revealed that RopA and RopB proteins formed fibrils in the capsules of the *R. leguminosarum* cells (Figure 6D,E) and extracellular material (Figure 6F,G) that bound anti-RopA or anti-RopB antibodies. Taken together, RopA and RopB form amyloid fibrils in vivo.

## 4. Discussion

The term “functional amyloid” was introduced in 2000 with discovery of the amyloid structure of hydrophobins—small proteins providing surface transition from a hydrophilic to a hydrophobic state through amphipathic membrane formation [68]. The first functional amyloid in bacteria, CsgA curlin which forms extracellular fimbriae called “curli”, was described in 2002 [21]. To date, numerous findings of different functional amyloids in bacteria and even archaea [19,20] demonstrated that these protein fibrils are widespread in the prokaryotic world, performing various biological functions that, despite being useful for bacteria, are harmful for their multicellular hosts [69,70]. In particular, probably the best characterized function of amyloids is their structural role in biofilm formation that is shared by various phylogenetically distant species of Gram-negative (Gammaproteobacteria) [69] and Gram-positive bacteria (Firmicutes) [71]. To date, up to 80% of bacterial infections are associated with biofilm formation [72,73]. Thus, despite the fact that pathological aggregation of human proteins causes about 50 diseases [1,74], the real number of amyloid-associated diseases is significantly higher since we should take into account those related with bacterial amyloid formation. This is a notable example of dualism in host–pathogen systems when amyloids are functional for bacteria but pathogenic for their host. Not only biofilm proteins but also toxins and different bacterial factors of virulence form amyloids that may be either inactivated for storage or functionally active [69]. Therefore, according to contemporary data, with the exception of several proteins regulating transcription [75,76,77], most bacterial amyloids are associated with the virulence and can be harmful for multicellular hosts promoting host–pathogen interactions.

The discovery of amyloid formation by RopA and RopB proteins of *R. leguminosarum* in this work suggests that bacterial amyloids may be involved not only in pathogenic but also in beneficial symbiotic interactions with multicellular hosts. We demonstrated that both full-length RopA and RopB form fibrils in vitro that possess resistance to ionic detergents and proteases, exhibit a high content of β-sheets, and bind ThT and CR in an amyloid-specific manner (Figure 2, Figure 3 and Figure 4). Thus, RopA and RopB are bona fide amyloid proteins. RopB amyloids exhibited higher resistance than RopA to treatment with proteases and persistent boiling in SDS-containing buffer (Figure 4C), similarly to CsgA curlin amyloids [21]. Different physicochemical characteristics of the RopA and RopB fibrils obtained in different buffers (Figure 2, Figure 3 and Figure 4) suggest that their high structural polymorphism may be important not only in vitro but also in vivo as in the case of prion (infectious amyloid) variants affecting their functions and properties [78]. Other examples of bacterial functional protein aggregates related to plant–microbial interactions are harpin amyloids from plant pathogen *X. axonopodis* that cause a hypersensitivity response in plants [28] and TasA protein from *Bacillus subtilis* [79]. TasA promotes rhizosphere colonization through involvement in biofilm formation and root attachment [80], but its structural identity with amyloids is controversial, representing a rather non-amyloid assembly of globular subunits without a characteristic cross-β structure [81].

Precise functions of RopA and RopB are still unknown, but these proteins are similar in terms of their primary structures to outer membrane porins which typically act as major virulence factors [82]. Here, we showed that full-length RopA and RopB formed bona fide amyloids in vitro (Figure 2, Figure 3 and Figure 4), aggregated when overexpressed in *S. cerevisiae*, assembled into amyloid fibrils at the surface of *E. coli* cells (Figure 5), and formed fibrils in vivo in the capsule of *R. leguminosarum* (Figure 6). Both these proteins existed in polymeric states in vivo, while monomers and dimers that might correspond to the membrane pores probably formed by these proteins occurred only in small amounts, mostly after boiling (Figure 6A). We cannot exclude that various bacterial proteins with bioinformatically predicted structural similarity with the outer membrane porins, in fact, represent extracellular amyloids.

The increase in expression of *ropA* and *ropB* in *R. leguminosarum* cells was previously demonstrated at the early stages of nodulation [66]. At the same time, the *ropA* gene becomes downregulated in the root nodule when *R. leguminosarum* release from the infection threads and transits from free-living cells to nitrogen-fixing bacteroids [83]. The *ropB* gene also seems to be switched off in bacteroids [84]. Thus, RopA and RopB are likely to be associated with the virulence of *R. leguminosarum*, and they act at the early stages of host plant colonization. Based on the data obtained, we hypothesize that RopA and RopB amyloid formation could be involved in the development of the early stages of plant–microbial symbiosis. Physiological relevance of this idea needs further clarification, but a certain increase in the amount and size of RopA aggregates was detected after addition of luteolin to the cultural media (Figure 5). Extracellular polysaccharides produced by different *Rhizobium* species are known to participate in their virulence, in particular, in the early recognition of bacteria by root cells [85]. Our data suggest that such virulence could be mediated not only by the polysaccharides but also by capsule-localized amyloids.

The repertoire of the *R. leguminosarum* amyloids may not be limited to RopA and RopB. In this work, using a PSIA assay, we identified 54 detergent-resistant proteins (Table S2, Supplementary Materials). A significant number of candidate proteins act as metabolic and DNA repair enzymes, and chaperonins. These proteins, such as DNA-helicase RuvB and 60-kDa chaperonin groL, participate in the formation of multicomponent protein complexes that, being detergent-resistant, are likely to be non-amyloidogenic. For example, resistance to treatment with ionic detergents was previously demonstrated for proteasome [33] and ribosome [86] proteins of *S. cerevisiae*. Interestingly, several ribosome proteins of *R. leguminosarum* (Alphaproteobacteria) are detergent resistant while the same proteins of *E. coli* (Gammaproteobacteria) are not resistant, suggesting structural differences between their ribosomes. Detergent-resistant protein fractions of *R. leguminosarum* are partially similar in terms of the functions of proteins comprising them to those previously identified by us in *E. coli* [36]. In particular, DNA helicases, chaperonins, and outer membrane porins were identified in both species (Figure 1, Table S2, Supplementary Materials). While most *R. leguminosarum* detergent-resistant proteins are cytoplasmic, 11 proteins were annotated as membrane proteins, including six porins and membrane transport proteins (Figure 1, Table S2, Supplementary Materials). Amyloid properties of transmembrane proteins were previously demonstrated for outer membrane proteins of *E. coli* OmpA (in vitro) [87] and OmpC (in vitro and, partially, in vivo) [88]. These proteins were also identified by PSIA in *E. coli* [36], and they share, with RopA and RopB, the predicted β-barrel structure and relation to virulence [89], as well as the OmpP2-like protein of *Mannheimia haemolytica* that was shown to form amyloid-like filaments [90]. Such β-barrel domains typical for porins of Gram-negative bacteria were predicted to be similar in terms of their sequence to amyloid-forming proteins, presupposing interdependence between them [91]. Notably, β-barrel domains may represent important structural amyloidogenic determinants not only of bacterial proteins but also eukaryotic proteins. For example, several seed storage proteins of plants called “cupins” that were predicted to be amyloidogenic [92] have similar β-barrel domains [93]. Detergent-resistant fractions of *R. leguminosarus* were also found to contain other virulence proteins, like NodT, which is probably involved in the secretion of nodulation factors [94]. This corresponds to the previously obtained bioinformatic data, which demonstrated that amyloidogenic regions in proteins of species belonging to the order Rhizobiale tend to co-occur with membrane or extracellular proteins, many of which are associated with virulence of these bacteria [95]. Overall, it becomes apparent that amyloids play an important role in the virulence of bacteria by promoting their interaction with a multicellular host.

## 5. Conclusions

Proteomic screening performed in this study revealed 54 potentially amyloidogenic detergent resistant proteins of the root-nodule bacterium *R. leguminosarum* bv. *viciae*. Some of them, including outer membrane proteins, are involved in the virulence of this species. Detailed analysis of the amyloid properties was performed for two selected candidates, RopA and RopB, which are similar in terms of their structure to the outer membrane porins and probably involved in the control of plant–microbial symbiosis. We found that full-length RopA and RopB form bona fide amyloid fibrils in vitro that possess resistance to treatment with ionic detergents and proteases, bind ThT, exhibit green birefringence upon staining with CR, and demonstrate a high content of β-sheets. The RopA and RopB proteins form intracellular aggregates in *S. cerevisiae* and extracellular amyloid fibrils in *E. coli.* In vivo, these proteins are localized in the congophylic and exhibiting green birefringence capsules of *R. leguminosarum* where they form fibrils. Taken together, we may conclude that RopA and RopB are novel amyloid proteins of *R. leguminosarum*.

## Supplementary Materials

The following are available online at https://www.mdpi.com/2218-273X/9/11/694/s1: **File S1**: Sequences of the *ropA* and *ropB R. leguminosarum* genes with the codon usage optimized for expression in *E. coli*; **Table S1**: List of primers used in this work; **Table S2**: Proteins identified in detergent-resistant fraction of *R. leguminosarum*; **Figure S1**: Mass spectrometry identification data of the RopA (OmpIIIA) and RopB proteins; **Figure S2**: Secondary structure of RopA and RopB in monomeric and aggregated states; **Figure S3**: Fluorescence of ThT bound to RopA and RopB in monomeric and aggregated states; **Figure S4**: Polarization microscopy of the CR-stained Sup35NM and Sup35M samples; **Figure S5**: Analysis of the protease resistance of the Sup35NM protein obtained in vitro at the initial point of incubation by Western blot.
