# Supplementary material for: Two Novel Amyloid Proteins, RopA and RopB, from the Root Nodule Bacterium Rhizobium leguminosarum
Source: Biomolecules. 2019 Nov 4;9(11):694. doi: 10.3390/biom9110694 (PMC6920782; doi:10.3390/biom9110694)
Supplement: Supplementary file 1 [file biomolecules-09-00694-s001.zip › Table S2.pdf]

Table S2: Proteins identified in detergent resistant fraction of *R. leguminosarum*

Gene	Protein	Accession[1]	Score[2]	Mw[3]	pI[4]	Sc[5]	Np[6]	Location[7]
ropA	Outer membrane protein IIIA	OM3A_RHILV	172.6	39.1	3.9	9.8	2	M
rpoB	DNA-directed RNA polymerase subunit beta	RPOB_RHIL3	93.9	153.6	4.9	12.5	16	C
mutS	DNA mismatch repair protein MutS	MUTS_RHIL3	67.5	98.6	5.3	13.7	10	C
acdS	1-aminocyclopropane-1-carboxylate deaminase	1A1D_RHILV	65	36.8	5.1	18.6	5	C
atpA	ATP synthase subunit alpha	ATPA_RHIL3	63.1	54.8	6	18.9	8	M
alaS	Alanine--tRNA ligase	SYA_RHIL3	60.6	95.4	5.2	14.1	12	C
mnmG	tRNA uridine 5-carboxymethylaminomethyl modification enzyme MnmG	MNMG_RHIL3	58.9	67.9	5.7	16.5	8	C
gltX	Glutamate--tRNA ligase	SYE_RHIL3	56.8	55.1	5.7	25.8	10	C
katG	Catalase-peroxidase	KATG_RHIL3	55.3	79.7	5.4	6.9	3	C
groL1	60 kDa chaperonin 1	CH601_RHIL3	55.1	57.9	4.9	24.9	10	C
tsf	Elongation factor Ts	EFTS_RHIL3	53.9	32.2	4.9	20.8	7	C
glnD	Bifunctional uridylyltransferase/uridylyl-removing enzyme	GLND_RHILV	51.5	106.7	6.9	9.5	8	C
dnaK	Chaperone protein DnaK	DNAK_RHIL3	51.1	68.5	4.7	22.6	11	C
infB	Translation initiation factor IF-2	IF2_RHIL3	50.7	99.6	7.9	8.8	7	C
trmD	tRNA (guanine-N(1)-)-methyltransferase	TRMD_RHIL3	50.2	26.6	5.8	30.3	5	C
hslV	ATP-dependent protease subunit HslV	HSLV_RHIL3	47.3	18.7	5.7	24.6	7	C
ilvD	Dihydroxy-acid dehydratase	ILVD_RHIL3	46.4	66	5.7	24.7	10	C
argG1	Argininosuccinate synthase 1	ASSY1_RHIL3	45.7	44.4	5.6	17.9	9	C
RL4503	UPF0235 protein RL4503	Y4503_RHIL3	45.5	11.1	9.7	62.1	6	N. d.

Gene	Protein	Accession[1]	Score[2]	Mw[3]	pI[4]	Sc[5]	Np[6]	Location[7]
ispDF	Bifunctional enzyme IspD/IspF	ISPDF_RHIL3	44.6	43.5	6	11.6	5	N. d.
modC	Molybdenum import ATP-binding protein ModC	MODC_RHIL3	42.8	38.8	7.9	14.6	4	M
rpoC	DNA-directed RNA polymerase subunit beta'	RPOC_RHIL3	42.3	156.3	6.6	10	13	C
prfA	Peptide chain release factor 1	RF1_RHIL3	41.4	40	4.9	28.7	8	C
miaB	(Dimethylallyl)adenosin e tRNA methylthiotransferase MiaB	MIAB_RHIL3	40	53.2	5.7	17.3	8	C
rplA	50S ribosomal protein L1	NDVA_RHIL3	39.6	24.1	10.1	29.2	6	C
ndvA	Beta-(1-->2)glucan export ATP-binding/permease protein NdvA	RL1_RHIL3	39.6	64.8	9.1	10.2	5	M
xseA	Exodeoxyribonuclease 7 large subunit	EX7L_RHIL3	39.2	57.4	10.4	18.6	8	C
glsA	Glutaminase	GLSA_RHIL3	37.8	33.1	6.9	19.4	5	C
pyrD	Dihydroorotate dehydrogenase (quinone)	PYRD_RHIL3	37.4	38.9	9.4	11.3	4	M
fhs	Formate--tetrahydrofolate ligase	FTHS_RHIL3	36.5	60.2	6.8	11.8	5	C
groL	60 kDa chaperonin	CH60_RHILV	35.9	57.8	5	25.5	10	C
recA	Protein RecA	RECA_RHILV	35.6	37.8	5.2	21.9	5	C
rbsA1	Ribose import ATP-binding protein RbsA 1	RBSA1_RHIL3	35.5	55.9	6.8	17	7	M
rplI	50S ribosomal protein L9	RL9_RHIL3	35.2	21.1	4.6	28.6	4	C
glyS	Glycine--tRNA ligase beta subunit	SYGB_RHIL3	35	77.3	5.6	9.9	6	C
proB	Glutamate 5-kinase	PROB_RHIL3	34.8	40.9	9.4	13.9	5	C
rbsA2	Ribose import ATP-binding protein RbsA 2	RBSA2_RHIL3	34.5	55.6	6.4	12.9	7	M

Gene	Protein	Accession[1]	Score[2]	Mw[3]	pI[4]	Sc[5]	Np[6]	Location[7]
ureC	Urease subunit alpha	URE1_RHIL3	34	61	5.6	4	4	C
accA	Acetyl-coenzyme A carboxylase carboxyl transferase subunit alpha	ACCA_RHIL3	33.9	34.6	6.1	21.8	4	C
ruvB	Holliday junction ATP-dependent DNA helicase RuvB	RUVB_RHIL3	33.9	38.4	5.3	23.7	6	C
purA	Adenylosuccinate synthetase	PURA_RHIL3	33.6	46.6	5.4	13	5	C
rplQ	50S ribosomal protein L17	RL17_RHIL3	33.4	15.3	11.1	21.4	5	C
rbsA3	Ribose import ATP-binding protein RbsA 3	RBSA3_RHIL3	32.9	55.5	6	6.2	4	M
rpsF	30S ribosomal protein S6	RS6_RHIL3	32.3	17.8	9.6	32.9	4	C
mutL	DNA mismatch repair protein MutL	MUTL_RHIL3	32	64.6	6.3	9.7	3	C
ropB	22 kDa outer membrane protein	MASZ_RHILV	31.9	22.5	4.2	9	2	M
glcB	Malate synthase G	ROPB_RHILV	31.9	80	5.6	9.4	8	C
ttcA	tRNA 2-thiocytidine biosynthesis protein TtcA	TTCA_RHIL3	31.5	32.8	6.9	12.1	5	C
tdh	L-threonine 3-dehydrogenase	TDH_RHIL3	31.2	37.8	5.9	20.9	5	C
nodT	Nodulation protein T	NODT_RHILV	30.9	51.5	6.6	11.2	5	M
hypD	Hydrogenase expression/formation protein HypD	HYPD_RHILV	30.8	42.6	6.5	6.2	3	C
betA	Oxygen-dependent choline dehydrogenase	BETA_RHIL3	30.8	0	6.9	6.2	2	M
leuC	3-isopropylmalate dehydratase large subunit	LEUC_RHIL3	30.6	51.1	5.6	19.6	8	C
groL	60 kDa chaperonin	CH60_RHIL3	30.5	57.3	4.9	14.2	7	C

[1]UniProt accession number;

[2]MASCOT protein score (index for determining accuracy identification by Mascot software);

[3]Calculated molecular weight. kDa;

[4]Calculated isoelectric point;

[5]Protein sequence coverage (%);

[6]Number of unique peptides matched to mass peaks;

[7]Protein location according to UniProt (https://www.uniprot.org/) database (M – membrane, C – cytoplasmic, n.d. – no data).
