# Supplementary material for: Two Novel Amyloid Proteins, RopA and RopB, from the Root Nodule Bacterium Rhizobium leguminosarum
Source: Biomolecules. 2019 Nov 4;9(11):694. doi: 10.3390/biom9110694 (PMC6920782; doi:10.3390/biom9110694)
Supplement: Supplementary file 1 [file biomolecules-09-00694-s001.zip › Fig S1.pdf]

A

Outer membrane protein IIIA OS=Rhizobium leguminosarum bv. viciae GN=ropA PE=1 SV=1 OM3A_RHILV MH+ (mono): 1.008 MH+ (avg): 1.008 Tolerance (Da): 5.500 Number of Peaks: 909

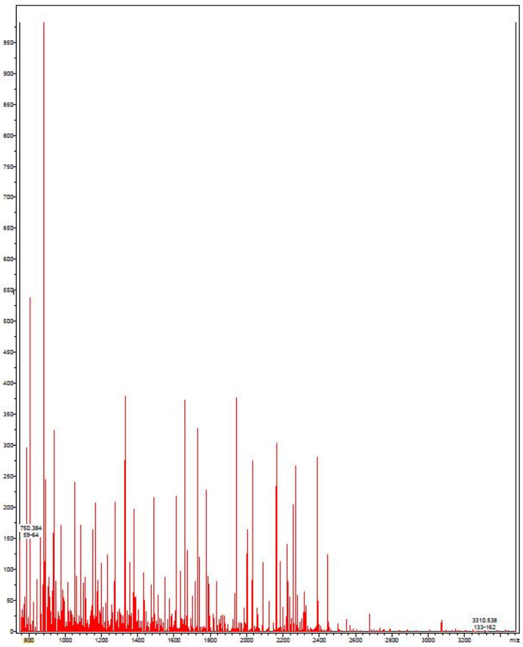

MNIRMVLLASAAAFAASTPVLAADAIVAAEPEPVEYVRVCDAYGTGYFYIPGTETCLK**IEGYIR**FQVNVGDNPGGDNDSDWDAVTAVRFSSRKSDTE
YGPLTGVIVMQFNADNASDQDAILDSAYLDVAGFR**AGLFYSWWDDGLSGETDDIGSVVTLHNSIR**YQYESGTFYAGLSVDELEDGVYQGTFTPGVI
PGTTDFTADDGPNNVGVAFGIGGTAGAFSYQVTGGWDVDNEDGAIRAMGTVEIGPGTFGLAGVYSSGPNSYYSSAEWAVAAEYAIKATDKLKITPGR
WHGHVPEDFDGLGDAWKVGLTVDYQIVENFYAKASVQYLDPQDGEDSTSGYFACSVRSNHLVDAPGLRIGSTTISF

B

22 kDa outer membrane protein OS=Rhizobium leguminosarum bv. viciae GN=ropB PE=3 SV=1 ROPB_RHILV MH+ (mono): 1.008 MH+ (avg): 1.008 Tolerance (Da): 5.500 Number of Peaks: 909

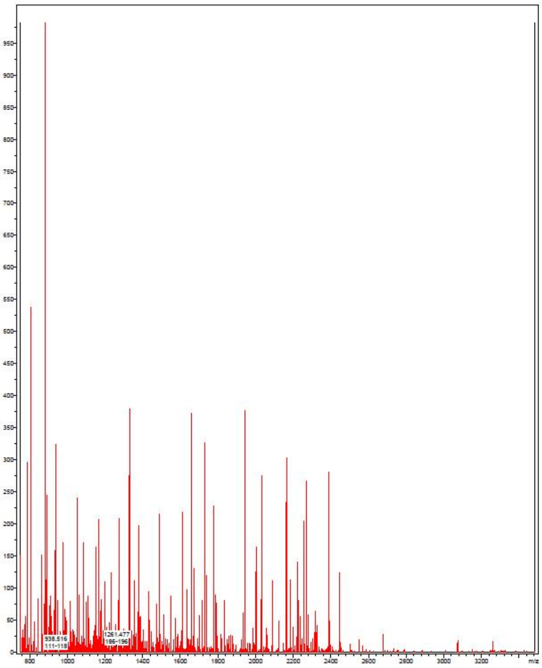

MRVLIAGLMASVFAIAGVSAAQAADRVDQVPEAPVAQEAPVKPAGSWEGFYLGGAGTYNMGDFGSDRHTYGFGGQVFTGYNWQQGQIVYGVESD
LGYSGDDVSSGGVENK**YGWNGSVR**GRVGYDMNPFLLYGTAGLAIGDVKVSDDTSDESKTNFGYTVGAGVEAFVTNNITTRLEYRYTDYQSK**DYDLD
SGSFSR**GYDENSVKLGIGVKF

Figure S1. Mass spectrometry identification data of the RopA (OmpIIIA) (A) and RopB (B) proteins. Amino acid sequences of the RopA (A) and RopB (B) proteins are shown. Peptides identified by mass spectrometry are indicated in yellow.
