# Supplementary material for: Two Novel Amyloid Proteins, RopA and RopB, from the Root Nodule Bacterium Rhizobium leguminosarum
Source: Biomolecules. 2019 Nov 4;9(11):694. doi: 10.3390/biom9110694 (PMC6920782; doi:10.3390/biom9110694)
Supplement: Supplementary file 1 [file biomolecules-09-00694-s001.zip › Fig S2.pdf]

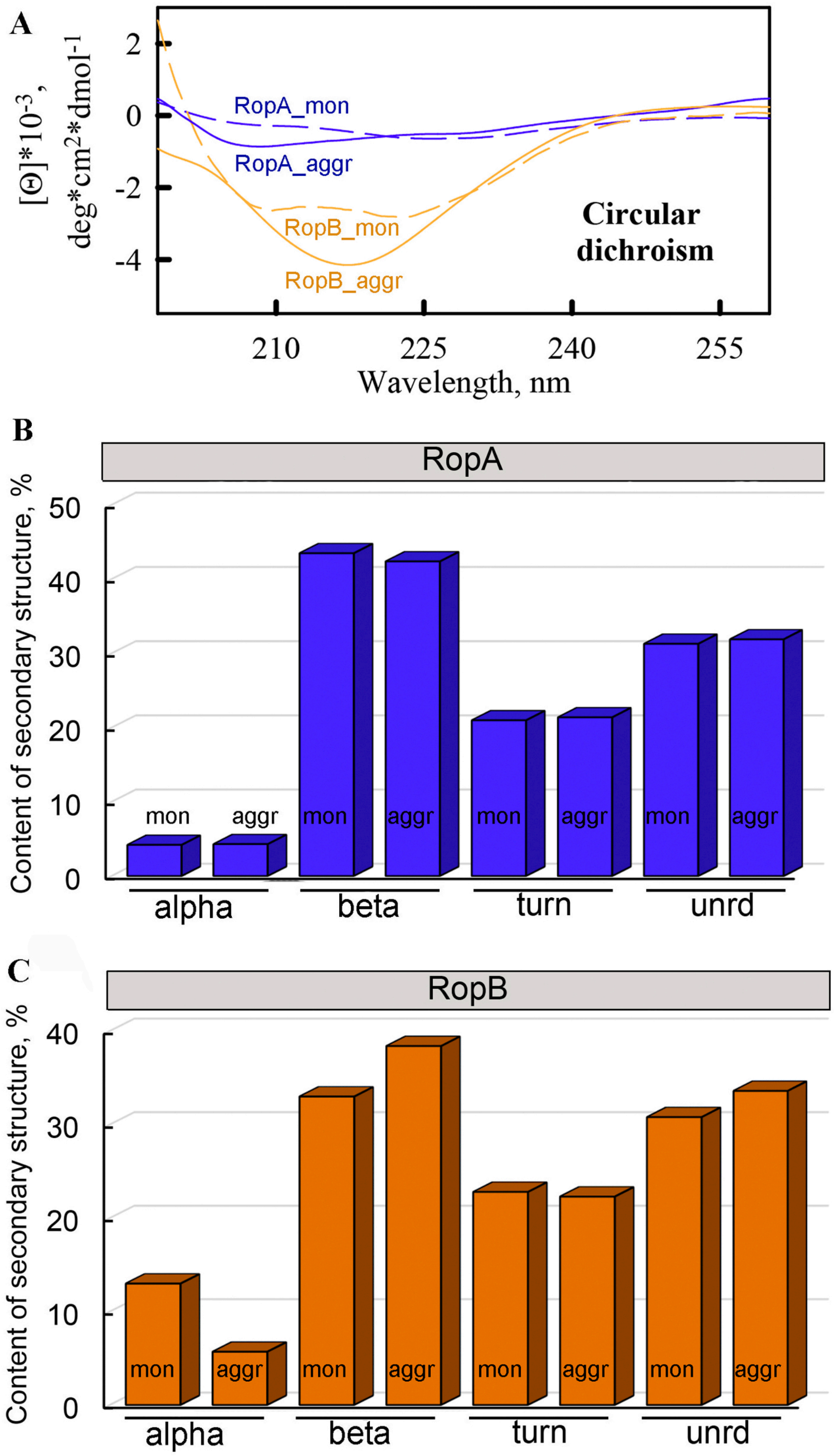

Figure S2. Secondary structure of RopA and RopB in monomeric and aggregated states. (A) far-UV circular dichroism (CD) spectra of monomeric RopA (blue dashed line) and RopB (orange dashed line) dissolved with the use of HFIP/water mixture and mature aggregates of RopA (blue solid line) and RopB (orange solid line). Figure shows content of elements of the secondary structure in (B) monomeric RopA (mon) and its aggregates (aggr) and in (C) monomeric RopB (mon) and its aggregates (aggr): alpha-helixes (alpha), beta-sheets (beta), turns (turn) and unordered (unrd) structure.
