# Supplementary material for: Two Novel Amyloid Proteins, RopA and RopB, from the Root Nodule Bacterium Rhizobium leguminosarum
Source: Biomolecules. 2019 Nov 4;9(11):694. doi: 10.3390/biom9110694 (PMC6920782; doi:10.3390/biom9110694)
Supplement: Supplementary file 1 [file biomolecules-09-00694-s001.zip › Fig S3.pdf]

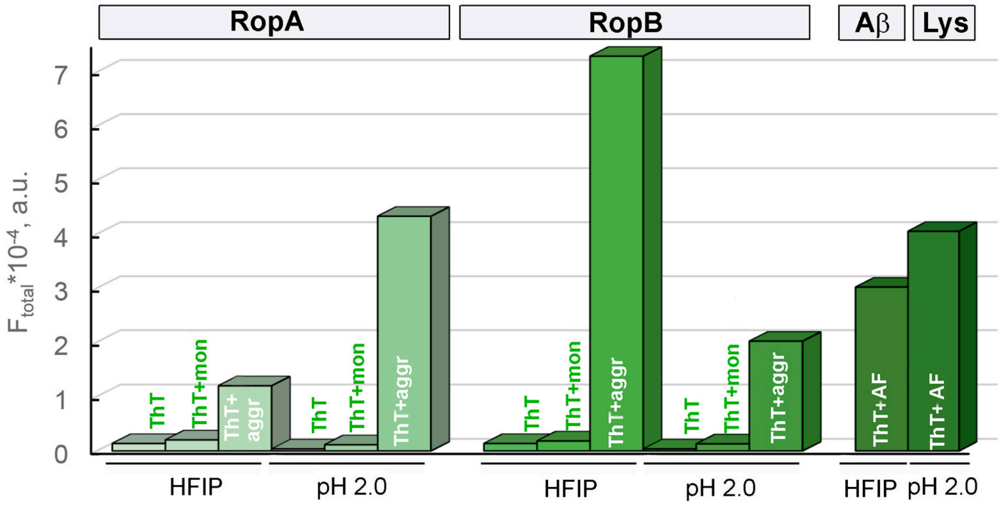

Figure S3. Fluorescence of ThT bound to RopA and RopB in monomeric and aggregated states. Figure shows corrected on the primary inner filter effect total fluorescence intensity of ThT in free state (ThT) in the conditions of fibrillogenesis (HFIP/water mixture (HFIP) and buffer with acidic pH 2.0, in the presence of RopA and RopB in monomeric (ThT+mon) and aggregated (ThT+aggr) states, in the presence of Aβ-peptide (1-42 aa) and lysozyme amyloid fibrils (ThT+AF) prepared using HFIP and acidic solution, respectively.
