# Supplementary material for: Two Novel Amyloid Proteins, RopA and RopB, from the Root Nodule Bacterium Rhizobium leguminosarum
Source: Biomolecules. 2019 Nov 4;9(11):694. doi: 10.3390/biom9110694 (PMC6920782; doi:10.3390/biom9110694)
Supplement: Supplementary file 1 [file biomolecules-09-00694-s001.zip › Fig S4.pdf]

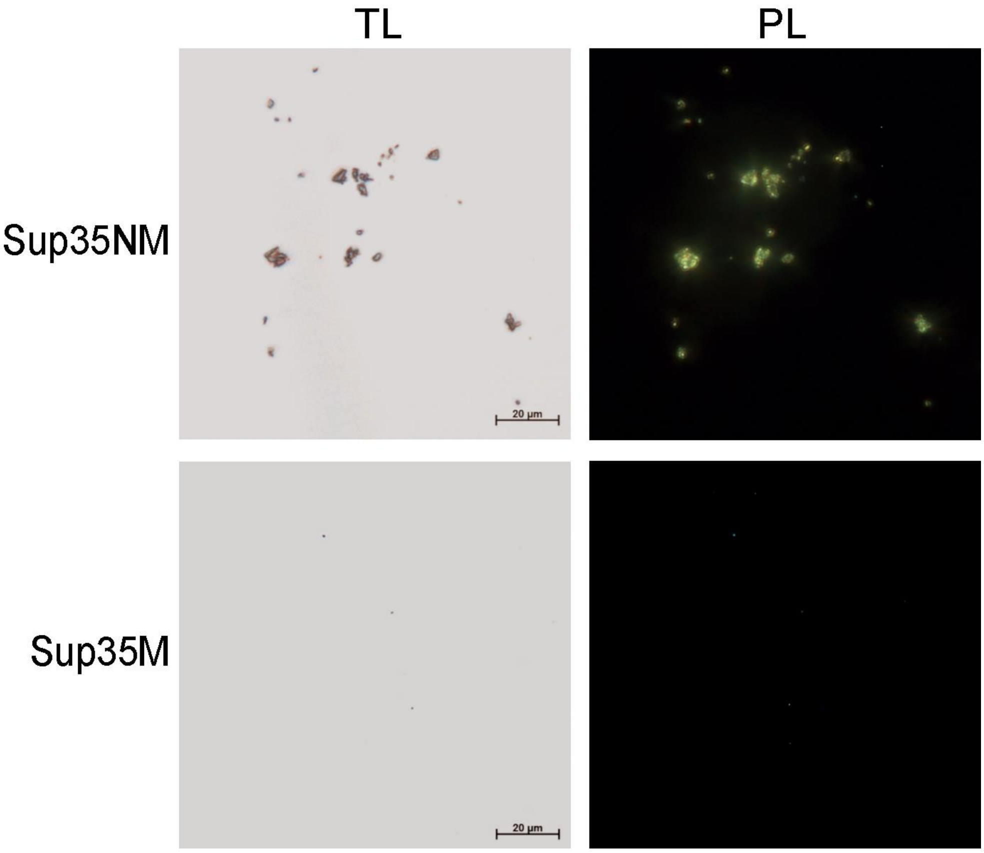

Figure S4. Polarized light microscopy of the CR-stained Sup35NM and Sup35M samples. TL – transmitted light, PL – polarized light. Scale bar is equal to 20 μm.
