# Supplementary material for: Two Novel Amyloid Proteins, RopA and RopB, from the Root Nodule Bacterium Rhizobium leguminosarum
Source: Biomolecules. 2019 Nov 4;9(11):694. doi: 10.3390/biom9110694 (PMC6920782; doi:10.3390/biom9110694)
Supplement: Supplementary file 1 [file biomolecules-09-00694-s001.zip › Fig S5.pdf]

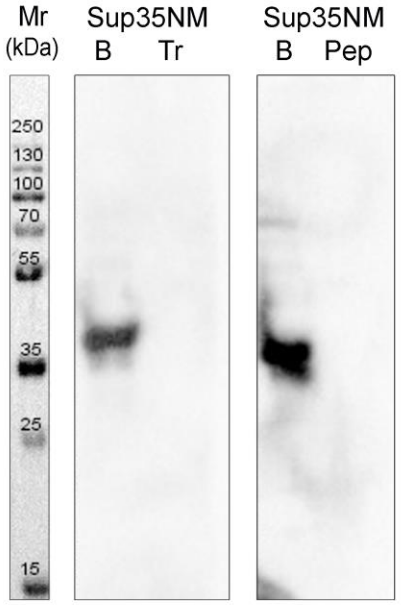

Figure S5. Analysis of the protease resistance of the Sup35NM protein obtained *in vitro* at the initial point of incubation by western-blot. B – samples boiled with 2% SDS; T – treated with trypsin and boiled with 2% SDS; P – treated with pepsin and boiled with 2% SDS. Corresponding molecular weights (kDA) are indicated.
