# Supplementary material for: Two Novel Amyloid Proteins, RopA and RopB, from the Root Nodule Bacterium Rhizobium leguminosarum
Source: Biomolecules. 2019 Nov 4;9(11):694. doi: 10.3390/biom9110694 (PMC6920782; doi:10.3390/biom9110694)
Supplement: Supplementary file 1 [file biomolecules-09-00694-s001.zip › File S1.pdf]

A

```
   1 M   A   D   A   I   V   A   A   E   P   E   P   V   E   Y   V   R   V   C   D
   1 ATGGCAGATGCGATTGTTGCAGCGGAACCGGAACCGGTGGAGTATGTTCGTGTGTGCGAT
  21 A   Y   G   T   G   Y   F   Y   I   P   G   T   E   T   C   L   K   I   E   G
  61 GCGTATGGCACTGGCTACTTCTACATTCCAGGCACCGAGACCTGTCTGAAGATTGAAGGC
  41 Y   I   R   F   Q   V   N   V   G   D   N   P   G   G   D   N   D   S   D   W
 121 TACATTCGCTTTCAGGTGAACGTTGGCGACAATCCAGGTGGCGACAACGATAGCGATTGG
  61 D   A   V   T   A   V   R   F   S   S   R   K   S   D   T   E   Y   G   P   L
 181 GATGCAGTGACTGCGGTTCGCTTCTCTAGCCGCAAGAGTGATACCGAGTATGGTCCACTG
  81 T   G   V   I   V   M   Q   F   N   A   D   N   A   S   D   Q   D   A   I   L
 241 ACTGGCGTGATTGTGATGCAGTTCAATGCGGATAACGCGAGCGATCAGGATGCGATTCTG
 101 D   S   A   Y   L   D   V   A   G   F   R   A   G   L   F   Y   S   W   W   D
 301 GATTCTGCGTATCTTGATGTTGCAGGCTTTCGTGCTGGCTTGTTCTATAGCTGGTGGGAT
 121 D   G   L   S   G   E   T   D   D   I   G   S   V   V   T   L   H   N   S   I
 361 GATGGCTTGAGTGGCGAAACCGATGACATTGGCTCTGTTGTGACCCTGCACAACAGCATT
 141 R   Y   Q   Y   E   S   G   T   F   Y   A   G   L   S   V   D   E   L   E   D
 421 CGCTATCAGTATGAATCAGGCACCTTCTATGCAGGTCTGAGCGTGGATGAACTGGAAGAT
 161 G   V   Y   Q   G   T   F   T   P   G   V   I   P   G   T   T   D   F   T   A
 481 GGCGTGTATCAAGGCACCTTTACTCCAGGTGTGATTCCGGGTACCACCGATTTTACTGCG
 181 D   D   G   P   N   N   V   G   V   A   F   G   I   G   G   T   A   G   A   F
 541 GATGATGGTCCGAACAACGTTGGCGTTGCGTTTGGCATCGGTGGCACCGCAGGCGCGTTT
 201 S   Y   Q   V   T   G   G   W   D   V   D   N   E   D   G   A   I   R   A   M
 601 AGCTATCAGGTGACTGGTGGCTGGGATGTGGACAACGAAGATGGCGCGATTCGTGCGATG
 221 G   T   V   E   I   G   P   G   T   F   G   L   A   G   V   Y   S   S   G   P
 661 GGCACCGTGGAGATTGGTCCAGGCACCTTTGGTCTTGCTGGCGTGTATAGCAGTGGCCCG
 241 N   S   Y   Y   S   S   A   E   W   A   V   A   A   E   Y   A   I   K   A   T
 721 AACAGCTACTATAGCTCAGCGGAATGGGCGGTTGCAGCGGAGTATGCGATCAAAGCGACC
 261 D   K   L   K   I   T   P   G   R   W   H   G   H   V   P   E   D   F   D   G
 781 GACAAACTGAAGATCACCCCAGGTCGCTGGCATGGCCATGTTCCGGAGGACTTTGATGGC
 281 L   G   D   A   W   K   V   G   L   T   V   D   Y   Q   I   V   E   N   F   Y
 841 TTAGGCGATGCGTGGAAAGTTGGCCTGACCGTGGACTATCAGATTGTGGAGAACTTCTAT
 301 A   K   A   S   V   Q   Y   L   D   P   Q   D   G   E   D   S   T   S   G   Y
 901 GCGAAAGCGTCTGTGCAGTATCTGGATCCGCAGGATGGCGAAGATAGCACCAGCGGCTAC
 321 F   A   C   S   V   R   S   N   H   L   V   D   A   P   G   L   R   I   G   S
 961 TTTGCGTGCTCAGTTCGCAGCAACCATCTGGTGGATGCTCCAGGTTTACGCATTGGCAGC
 341 T   T   I   S   F   Stop
1021 ACCACCATCAGCTTCTAA
```

B

```
   1 M   A   D   R   V   D   Q   V   P   E   A   P   V   A   Q   E   A   P   V   K
   1 ATGGCAGATCGTGTGGATCAGGTACCGGAAGCACCGGTTGCTCAAGAAGCACCAGTGAAA
  21 P   A   G   S   W   E   G   F   Y   L   G   G   A   G   T   Y   N   M   G   D
  61 CCAGCAGGCAGCTGGGAAGGCTTCTATCTTGGTGGTGCTGGCACCTACAACATGGGTGAC
  41 F   G   S   D   R   H   T   Y   G   F   G   G   Q   V   F   T   G   Y   N   W
 121 TTTGGCTCTGATCGTCACACCTATGGCTTTGGAGGTCAGGTGTTCACTGGCTACAACTGG
  61 Q   Q   G   Q   I   V   Y   G   V   E   S   D   L   G   Y   S   G   D   D   V
 181 CAGCAAGGCCAGATTGTGTATGGTGTGGAGAGCGATCTTGGCTACAGTGGCGATGATGTG
  81 S   S   G   G   V   E   N   K   Y   G   W   N   G   S   V   R   G   R   V   G
 241 AGCTCTGGTGGAGTGGAGAACAAGTATGGCTGGAATGGCAGCGTTCGTGGTCGTGTTGGC
 101 Y   D   M   N   P   F   L   L   Y   G   T   A   G   L   A   I   G   D   V   K
 301 TATGACATGAATCCGTTTCTGCTGTATGGCACTGCAGGCTTAGCGATTGGCGATGTGAAA
 121 V   S   D   D   T   S   D   E   S   K   T   N   F   G   Y   T   V   G   A   G
 361 GTGAGCGATGACACCAGCGATGAGAGCAAGACCAACTTTGGCTATACCGTTGGTGCAGGC
 141 V   E   A   F   V   T   N   N   I   T   T   R   L   E   Y   R   Y   T   D   Y
 421 GTGGAAGCGTTTGTGACCAACAACATCACCACTCGCTTAGAGTATCGCTACACCGACTAT
 161 Q   S   K   D   Y   D   L   D   S   G   S   F   S   R   G   Y   D   E   N   S
 481 CAGAGCAAAGACTATGATCTGGACAGTGGCAGCTTTTCTCGTGGCTATGATGAGAACAGC
 181 V   K   L   G   I   G   V   K   F   Stop
 541 GTGAAACTTGGCATTGGCGTGAAGTTCTAA
```

File S1. Sequences of the *ropA* (A) and *ropB* (B) *R. leguminosarum* genes with the codon usage optimized for expression in *E. coli.*
