# Supplementary material for: Two Novel Amyloid Proteins, RopA and RopB, from the Root Nodule Bacterium Rhizobium leguminosarum
Source: Biomolecules. 2019 Nov 4;9(11):694. doi: 10.3390/biom9110694 (PMC6920782; doi:10.3390/biom9110694)
Supplement: Supplementary file 1 [file biomolecules-09-00694-s001.zip › Table S1.pdf]

Table S1: List of primers used in this work.

Primer	Sequence 5′-3′
FHindRopA	TACCAAGCTTATGGCAGATGCGATTGTTGCAG
RBglRopA	ATGAAGATCTGAAGCTGATGGTGGTGCTG
FHindRopB	TACCAAGCTTATGGCAGATCGTGTGGATCAGG
RBamRopB	AGATGGATCCGAACTTCACGCCAATGCCAAGT
FAlicRopA	AGAAGGAGATATAACTATGGCAGATGCGATTGTTGCAG
RAlicRopA	GTGGTGGTGATGGTGATGGCCGAAGCTGATGGTGGTGCTG
FAlicRopB	AGAAGGAGATATAACTATGGCAGATCGTGTGGATCAGG
RAlicRopB	GTGGTGGTGATGGTGATGGCCGAACTTCACGCCAATGCCAAGT
